# The neck as a keystone structure in avian macroevolution and mosaicism

**DOI:** 10.1186/s12915-023-01715-x

**Published:** 2023-10-13

**Authors:** Ryan D. Marek, Ryan N. Felice

**Affiliations:** 1 Centre for Integrative Anatomy, Department of Cell and Developmental Biology, University College London, London, UK; 2 Department of Life Sciences, Natural History Museum, London, UK; 3 Department of Genetics, Evolution, and Environment, University College London, London, UK

**Keywords:** Avian, Neck, Axial skeleton, Mosaic, Integration

## Abstract

**Background:**

The origin of birds from non-avian theropod dinosaur ancestors required a comprehensive restructuring of the body plan to enable the evolution of powered flight. One of the proposed key mechanisms that allowed birds to acquire flight and modify the associated anatomical structures into diverse forms is mosaic evolution, which describes the parcelization of phenotypic traits into separate modules that evolve with heterogeneous tempo and mode. Avian mosaicism has been investigated with a focus on the cranial and appendicular skeleton, and as such, we do not understand the role of the axial column in avian macroevolution. The long, flexible neck of extant birds lies between the cranial and pectoral modules and represents an opportunity to study the contribution of the axial skeleton to avian mosaicism.

**Results:**

Here, we use 3D geometric morphometrics in tandem with phylogenetic comparative methods to provide, to our knowledge, the first integrative analysis of avian neck evolution in context with the head and wing and to interrogate how the interactions between these anatomical systems have influenced macroevolutionary trends across a broad sample of extant birds. We find that the neck is integrated with both the head and the forelimb. These patterns of integration are variable across clades, and only specific ecological groups exhibit either head-neck or neck-forelimb integration. Finally, we find that ecological groups that display head-neck and neck-forelimb integration tend to display significant shifts in the rate of neck morphological evolution.

**Conclusions:**

Combined, these results suggest that the interaction between trophic ecology and head-neck-forelimb mosaicism influences the evolutionary variance of the avian neck. By linking together the biomechanical functions of these distinct anatomical systems, the cervical vertebral column serves as a keystone structure in avian mosaicism and macroevolution.

**Supplementary Information:**

The online version contains supplementary material available at 10.1186/s12915-023-01715-x.

## Background

The ecological diversity of modern birds is almost unmatched among terrestrial vertebrates [1]. Understanding how birds achieved such hyperdiversity in ecology, morphology, and locomotor behavior is a key question in macroevolution and macroecology. Several putative drivers of this ecological and morphological hyperdiversity have been proposed, including the acquisition of flight [2], the presence of cranial kinesis [3, 4] or adaptations in the hindlimb structure and function [5]. However, it is clear that no single key innovation can explain this clade’s anatomical variation. Rather, birds are the archetypical example of mosaic evolution. Mosaic evolution describes the combined presence of ancestral and derived traits within a single organism and is achieved when traits evolve at heterogeneous rates and modes [6]. Compared to non-avian theropod dinosaurs, birds are thought to exhibit increased mosaicism in the post-cranial skeleton thanks to increased modularity (i.e. semi-independent variation) in the forelimb, hindlimb and tail [7]. This foundational hypothesis has been used to explain the morphofunctional diversification of the avian skeleton [8–10]. Despite the ubiquity of the mosaic evolution hypothesis in bird evolution, avian mosaicism is often studied in relation to specific anatomical systems, such as the skull [11] or appendicular skeleton [8, 9]. In contrast, the axial skeleton has largely been excluded from the study of mosaic evolution in birds, despite the profound importance of the axial skeleton in locomotor mechanics and the numerous synapomorphic features of the avian spine [12–15].

Reorganization of the axial column has promoted diversity elsewhere in Vertebrata—shifts in the patterning and regionalization [16–23], as well as the integration of vertebrae with other skeletal elements [24, 25] has underpinned key evolutionary radiations and successes across the clade. Birds display a highly derived and variable axial skeleton [12–15], with the elongated, S-shaped cervical region as perhaps its most apparent feature. Morphological diversity within the avian neck is unparalleled amongst extant vertebrates—the number of cervical vertebrae can vary between 10 and 26 [15]. This has led to an equally unrivalled functional diversity within the avian neck, whereby the neck contributes to many habitual behaviors—from feeding and prey capture [26–29] to tool use and, remarkably, active involvement in tripedal locomotion in some species of parrot [30]. This morpho-functional diversity contrasts with the relative conservatism of the neck in closely related non-avian theropod dinosaurs, which is predominantly adapted for carnivory and prey capture [31, 32]. With the neck displaying such a variety of forms and functions in birds, it is clearly an important aspect of avian biology, yet we currently do not understand the tempo and mode of its evolution nor how cervical skeletal evolution fits in the mosaic of bird evolution more broadly (although some work exists on the potential integration between the neck and hindlimb [33]).

We hypothesize that the correlations between the cervical spine and other related anatomical systems play an important role in shaping the evolutionary dynamics of this structure. The grasping capability of the theropod forelimb was lost during the evolution of the flight-capable wing, and it is often hypothesized that the avian neck acts as a ‘surrogate forelimb’ in tandem with the beak to provide extant avians a method to manipulate their surroundings [4, 9, 15]. This ‘surrogate forelimb’ hypothesis not only provides an explanation as to why the avian neck displays such a diverse morpho-functional signal among vertebrates, but also suggests that the avian neck and forelimb may be integrated and have co-evolved during the evolution of powered flight [15]. The other major constituent of the pre-thoracic skeleton is the head, the mass of which is a universal constraint on neck morphology and construction across vertebrates [34, 35] and must be considered when considering avian neck evolution. Here, we investigate the patterns of integration between the neck, forelimb and head, as well as the rate of cervical morphological evolution across 112 species of extant birds (Additional file 1: Table S1) to understand the tempo and mode of avian cervical evolution and how the axial column contributes to our understanding of avian macroevolution.

## Results

### Allometric, phylogenetic and ecological drivers of morphological variation in avian cervical vertebrae

As the number of cervical vertebrae varies between species, we established a ‘functional homology’ between species by studying the second cervical vertebrae (C2) and vertebrae at 25%, 50% and 75% along the cervical column (herein referred to as C25%, C50% and C75%, respectively), as well as the last cervical vertebrae [36, 37]. We studied the phylogenetic, allometric and ecological signal of vertebral shape at multiple anatomical scales: at the level of individual regions and at the level of the whole neck (referred to herein as the ‘pooled’ dataset, where all regions are investigated together). Head-neck and neck-forelimb integration were investigated at these same anatomical levels—at the level of the individual vertebrae and at the level of the whole neck (pooled dataset). We further investigated how these patterns differ across dietary and foraging guilds. We quantified multivariate phylogenetic signal (*K*_mult_) for each of the studied vertebral regions, as well as for a pooled dataset that represented vertebral morphology across the entire neck (Additional file 1: Table S2). The influence of phylogeny is significant, with the last cervical vertebrae displaying the lowest phylogenetic signal (*K*_mult_ = 0.694, Additional file 1: Table S2) and the vertebrae at 25% along the cervical column (C25%) displaying the highest phylogenetic signal (*K*_mult_ = 1.156, Additional file 1: Table S2). We tested the effect of allometry and ecology on the morphological variation of cervical vertebrae using phylogenetic permutational multivariate analyses of variance (MANOVAs) [38]. Body mass has a significant but weak correlation with vertebral morphology across the pooled dataset (*p* = 0.04, *R*^2^ = 0.016, Additional file 1: Table S3). Body mass does not significantly correlate with vertebral morphological variation in three out of the five studied vertebrae (C2, C25% and C75%) but retains significance in C50% (*p* = 0.029, Additional file 1: Table S3) and the last cervical vertebrae (*p* = 0.005, Additional file 1: Table S3); however, the correlation coefficient is weak (*R*^2^ = 0.018 and 0.019, Additional file 1: Table S3). Variation in vertebral morphology within the pooled dataset is significantly related to foraging style (*p* = 0.014, *R*^2^ = 0.103, Additional file 1: Table S3) but not dietary guild. When repeated for individual cervical regions, the foraging style was found to be significantly correlated with variation in vertebral morphology in the last cervical vertebrae (*p* = 0.007, *R*^2^ = 0.107 Additional file 1: Table S3), and dietary guild displays a significant correlation in C75% (*p* = 0.024, *R*^2^ = 0.109 Additional file 1: Table S3).

### The role of neck-forelimb and head-neck integrations in avian cervical evolution

Total neck morphology is significantly integrated with both the head and the forelimb (*p* < 0.05, Additional file 1: Table S4). Head-neck and neck-forelimb integrations are similar in strength, as we compared effect sizes for separate phylogenetic two-block partial least squares (2BPLS) analyses and recovered no significant differences between the strength of these integration patterns (neck-forelimb integration was significantly stronger than head-neck integration in the last cervical vertebrae for none-size corrected data, Additional file 1: Table S4).

**Fig. 1:**
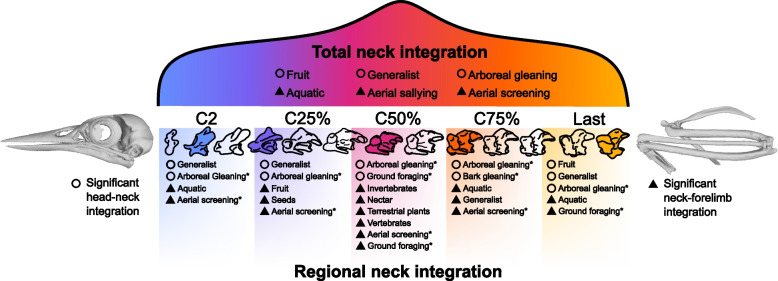
Diagrammatic representation of significant integration between the neck, head and forelimb across extant Aves. Circles represent significant integration between neck vertebral morphology and the head; triangles represent significant integration between neck vertebral morphology and the forelimb. ‘Total neck’ refers to all five vertebral regions entered into a 2BPLS together. Asterisks denote foraging guild; a lack of asterisks denote dietary guild

These results are variable across birds. Only specific ecological groups repeatedly display a significant neck-forelimb and/or head-neck integration (Fig. 1, Additional file 1: Tables S5). Neck-forelimb integration occurs across more dietary guilds than head-neck integration (11 guilds versus 6 guilds, respectively, Fig. 1, Additional file 1: Table S5). Aquatic feeders frequently (total group, C2, C75%, last cervical vertebrae) exhibit significant neck-forelimb integration and a lack of head-neck integration (Fig. 1, Additional file 1: Table S5). Other dietary guilds display significant neck-forelimb integration, but only in singular areas of the cervical spine (Fig. 1, Additional file 1: Table S5), such as granivores (C25%), insectivores (C50%), nectivores (C50%), terrestrial herbivores (C50%, *n* = 5) and carnivores (C50%, *n* = 4). Frugivores and generalist feeders exhibit both head-neck and neck-forelimb integration; however, both groups more commonly exhibit significant integration between head and specific vertebrae than between the forelimb and specific vertebrae (generalists 3 instances of head-neck integration versus 1 neck-forelimb integration and 2 versus 1 for frugivores, Fig. 1, Additional file 1: Table S5) across the cervical spine. There is only one instance whereby the two integration regimes co-occur, in the middle (C50%) vertebrae of generalists (Fig. 1, Additional file 1: Table S5).

**Fig. 2:**
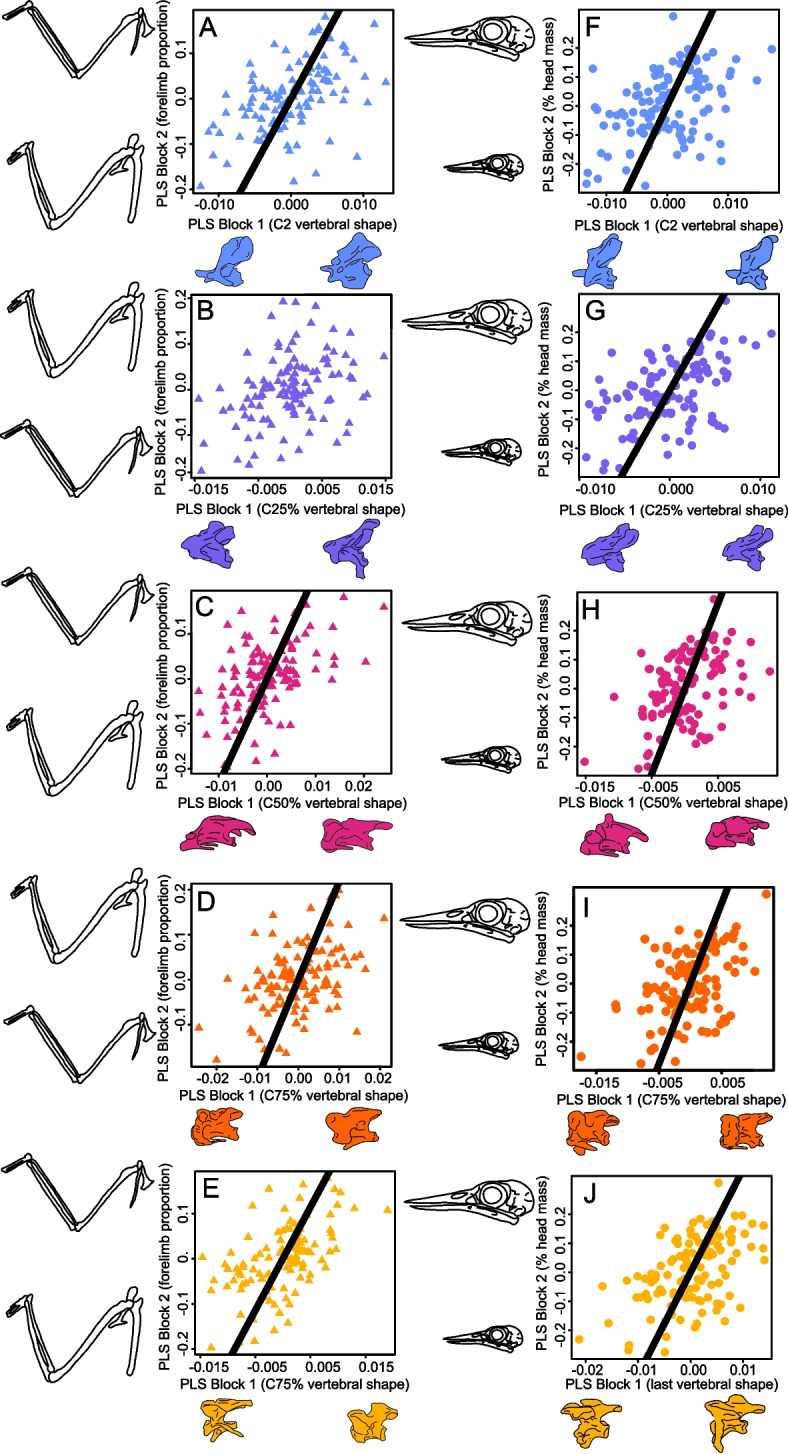
2BPLS plots of vertebral shape versus forelimb proportion (**A**–**E**) and percentage head mass (**F**–**J**). In C2 (**A**) and the last cervical vertebrae (**E**), elongation of distal forelimb elements is associated with increased intervertebral stability (decreases to centrum length and neural spine height). In the middle cervical vertebrae (**B**–**D**), longer distal forelimb elements are correlated with increases to intervertebral flexibility (increased centrum length, decreased neural spine height). Increases in relative head mass correspond to vertebral shape changes associated with intervertebral flexibility in C2-C50% (**F**–**H**) and increased stability in C75% and the last cervical vertebrae (**I**, **J**). The drawings below the *X*-axes display the vertebral shape change between end members along PLS block 1 for each individual cervical region for neck-forelimb (**A**–**E**) and head-neck (**F**–**J**) integration. In **A**–**E**, *Y*-axis drawings depict forelimb proportion change for end members across PLS block 2 for each individual cervical region. In **F**–**J**, *Y*-axis drawings display purely diagrammatical changes in head mass to visually display a change in head mass across the *Y*-axes. Representative species for end-member morphology for PLS block 1 are *Topaza pyra* and *Grus leucogeranus* (**A**), *Phoebastria irrorata* and *Spheniscus mendiculus* (**B**), *Topaza pyra* and *Phoebastria irrorata* (**C**), *Sula dactylatra* and *Topaza pyra* (**D**), *Topaza pyra* and *Grus leucogeranus* (**E**), *Calyptomena viridis* and *Heleia goodfellowi* (**F**), *Lipaugus vociferans* and *Zavattariornis stresemanni* (**G**, **H**, **J**) and *Capito niger* and *Glareola pratincola* (**I**). Representative end-member species for morphology for PLS block 2 are *Phalacrocorax harrisi* and *Sula dactylatra*

For C2 and the most proximal cervical vertebra, elongation of distal forelimb elements (radius, ulna, carpometacarpus) relative to proximal elements (coracoid, scapula, humerus—Fig. 2A–E; Additional file 1: Table S5) is associated with decreased intervertebral flexibility (decreases to centrum length and increases to neural spine height) [39, 40]. The opposite pattern is observed at the C25% and C75% positions, with longer distal forelimb elements correlated with longer centrum length and inferred intervertebral flexibility. The C50% vertebra follows the same pattern as C25% and C75%, with a positive correlation between the length of the centrum and the length of the autopodium + zeugopodium relative to the stylopodium (note that because the sign of partial least-squares, PLS, scores and loadings are arbitrary, the direction appears reversed in C50% but the pattern is the same). The 2BPLS further reveals increases in partial least-squares (PLS) scores across all cervical vertebrae are associated with increases in relative head mass (body mass adjusted, Fig. 2F–J, Additional file 1: Table S5). The vertebral shape changes associated with this increase in relative head mass are related to increased intervertebral flexion in the distal and middle vertebrae (C2–C50%, Fig. 2) and increased stability in the more proximal vertebrae (C75% and the last cervical vertebrae, Fig. 2).

The number of incidences of each integration pattern (neck-forelimb and head-neck) within specific foraging guilds is equal, with each integrative relationship occurring within 11 guilds (Fig. 1, Additional file 1: Table S5). Neck-forelimb integration is universally present across all cervical regions and never co-occurs with head-neck integration, except within the middle vertebrae of ground-foraging birds (Fig. 1, Additional file 1: Table S5). The pooled vertebral dataset of aerial sallying and aerial screening birds displays a significant neck-forelimb integration, but this relationship is never significant within any individual cervical region for aerial sallyers (Fig. 1, Additional file 1: Table S5). Ground foragers exhibit significant neck-forelimb integration in C50% and the last cervical vertebrae, and in C50%, this co-occurs with significant head-neck integration (Fig. 1, Additional file 1: Table S5). Head-neck integration is significant in all studied vertebral regions for arboreal gleaning birds and solely in C75% for bark gleaners (Fig. 1, Additional file 1: Table S5).

Previous studies have noted the importance of the aquatic lifestyle and carnivorous feeding on avian functional morphology [15, 33, 41]; thus, we performed additional tests to examine the patterns of cervical integration between these groups. Significant neck-forelimb integration is again recovered more frequently than head-neck integration, with significant head-neck integration only present in the last cervical vertebrae of non-carnivorous birds (Additional file 1: Table S5). Neck-forelimb integration is significant in carnivorous birds across the pooled dataset, C75%, and in the last cervical vertebrae. Likewise, in non-carnivorous birds, neck-forelimb integration is significant also C25% through to the last cervical vertebrae (Additional file 1: Table S5). Neck-forelimb integration is again more frequently found across the cervical spine when the data is split by water habitation (10 instances of significant neck-forelimb integration versus 3 head-neck, Additional file 1: Table S5). Aquatic birds display significant neck-forelimb integration within the pooled dataset, C2, C75%, and in the last cervical vertebrae and exhibit significant head-neck integration exclusively in C2 (Additional file 1: Table S5). Non-aquatic birds display significant neck-forelimb integration across all vertebral groups studied, and significant head-neck integration in only C2 and the last cervical vertebrae (Additional file 1: Table S5). There were no significant differences between the strength of head-neck and neck-forelimb integration across any of these antonymous groups (Additional file 1: Table S5).

### Tempo of avian cervical evolution

**Fig. 3:**
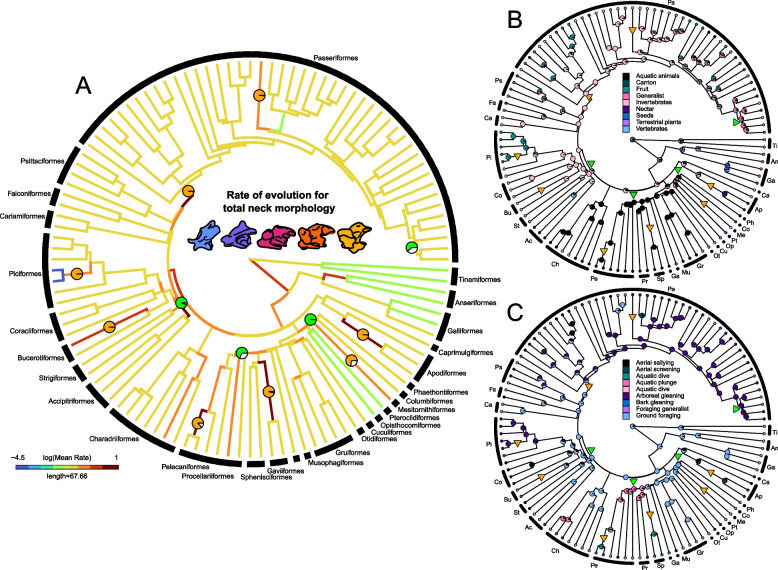
Shifts in evolutionary rates of total neck morphology and stochastic character maps of ecological characters. **A** Evolutionary rates of total neck morphology estimated using BayesTraitsV3 using a variable-rate model and lambda tree transformation, along with significant shifts in the rates of nodes (green pies) and branches (orange pies); pie charts denote the posterior probability of a rate shift occurring at that location. **B**, **C** Stochastic character maps of dietary (**B**) and foraging (**C**) guild, with rate shifts from **A** indicated by triangles (node shifts represented in green, branch shifts represented in orange). The trees in **A**–**C** all display the same orientation, and so clade names are abbreviated in **B** and **C**

**Fig. 4:**
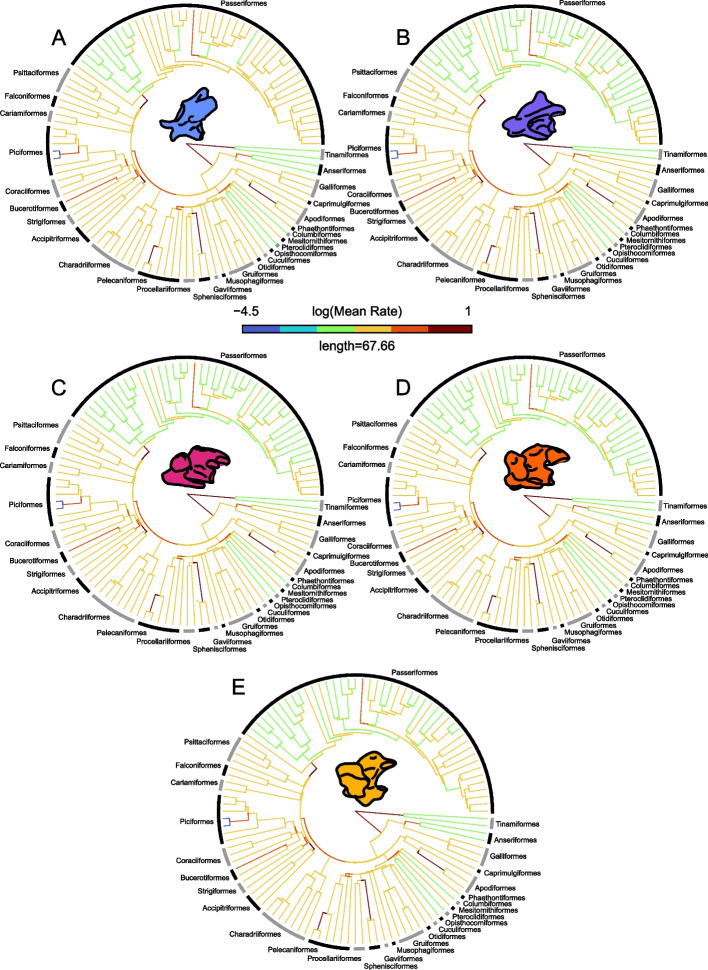
Evolutionary rates of vertebral morphology for avian cervical **A** C2 vertebrae, **B** C25% vertebrae, **C** C50% vertebrae, **D** C75% vertebrae and **E** last vertebrae

We fit several alternative evolutionary models to our data, including Brownian motion, lambda, kappa and delta with variable rates of evolution. For all vertebrae combined as well as for each individual cervical region, the variable-rate lambda model had the highest marginal likelihood (∆ Bayes factor > 10, Additional file 1: Table S6). Evolutionary rates across the entire neck are fastest along the stem of highly specialized clades such as Trochilidae, Phalacrocoracidae, Accipitriformes, Sphenisciformes and at the very base of Passeriformes (Fig. 3A). Rates are slowest within Palaeognathae, Anseriformes and at the base of derived members of Sylviidae (Fig. 3A). The patterns of rates of phenotypic evolution are largely similar for the cervical column as a whole and individual cervical segments (Fig. 4). Suboscines display lower rates in C2, there are generally slower rates across Passeriformes in C25% through C75% (however high rates are retained in Paridae) and Anseriformes exhibit a higher rate of evolution in C75% and in the last cervical vertebrae (Fig. 4).

We then calculated the rates of neck evolution for each dietary and foraging group—a multi-rate model was better supported than a single-rate model for both dietary and ecological groupings across all cervical regions (Additional file 1: Table S6). Generalist feeders, along with frugivores and insectivores, exhibit the highest evolutionary rate, whereas carnivores (*n* = 4) and carrion feeders (*n* = 3) exhibit the lowest rate amongst the dietary groups (Additional file 1: Tables S7, S8). Among foraging guilds, ground foragers and arboreal gleaners have the fastest evolutionary rates, and aquatic surface foragers (*n* = 1) along with generalist foragers have the slowest rates (Additional file 1: Tables S7, S8). A multi-rates model was also best supported for flesh versus non-flesh feeders and for aquatic dwelling versus non-aquatic dwelling taxa (Additional file 1: Table S6). Flesh feeders (Additional file 1: Tables S7, S8) and aquatic dwelling taxa both displayed lower rates of evolution (Additional file 1: Tables S7, S8). Rates of evolution are significantly different between the vertebral regions (Additional file 1: Table S9), with the fastest rate found in the last cervical vertebrae and the slowest in the middle vertebrae (C50%, Additional file 1: Table S9).

We identified significant shifts in the rate of total neck evolution at nodes and branches (i.e. those with a posterior probability of > 50%) and mapped them onto the phylogeny (Fig. 3A) alongside separate ecological stochastic character maps for dietary and foraging guilds (Fig. 3B, C). This allowed us to identify any co-occurrences between significant rate shifts in total neck morphology and the presence of either head-neck or neck-forelimb integration. A total of 12 significant rate shifts in whole neck vertebral morphology were found, 4 of which were positioned at nodes and 8 were positioned on branches (Fig. 3A). All but one of these rate shifts are situated at the nodes and branches which display elevated rates of morphological evolution (Fig. 3A). Nine out of 12 significant shifts were associated with ecological groups which displayed either significant head-neck or neck-forelimb integration at the level of the whole neck (Fig. 1, 3B, C). Significant rate shifts in total neck morphology are mostly observed in birds that feed on aquatic animals (3 rate shifts) that display significant neck-forelimb integration, as well as birds that forage via arboreal gleaning (4 rate shifts) and generalist feeders (2 rate shifts) that both display significant head-neck integration (Fig. 1, 3B, C).

## Discussion

The avian neck is significantly integrated with both the head and the forelimb (Additional file 1: Table S4). We also find that the strength of head-neck and neck-forelimb integration is equal (Additional file 1: Table S4) and that each pattern of integration is only found in specific ecological groups of birds (Fig. 1, Additional file 1: Table S5). Morphological variation of cervical vertebrae is significantly associated with ecology; however, this association is somewhat weak (Additional file 1: Table S3). Rates of neck evolution vary greatly across Aves with the highest occurring within highly specialized clades (Trochilidae, Sphenisciformes and Accipitriformes) and the lowest occurring within Paleognathae, Anseriformes and Passeriformes (Fig. 3A). Differences in the rate of neck evolution were also found between regions of the cervical spine, with the fastest rates occurring in the cervical region that displayed the highest degree of integration, the last cervical vertebrae (Additional file 1: Tables S7-S9). Twelve significant shifts in the rate of total neck morphology were found across the dataset (Fig. 3A), 9 of which were associated with groups that displayed either significant head-neck or neck forelimb integration (Fig. 3C).

A long-standing question in evolutionary biology is whether phenotypic integration constrains or facilitates the evolution of morphological diversity [42–44]. Integration is thought to facilitate phenotypic change when the direction of selection aligns with the major axis of variation of the integrated phenotype [42, 45]. Since both integration regimes are associated with rate shifts at nodes and branches that display relatively fast rates of evolution, it appears that selective forces for head, neck and forelimb morphology are complementary. As head-neck and neck-forelimb integration very rarely co-occur within any particular avian group, it appears that the major axis of variation of neck morphology is trending separately in the same direction as head and forelimb morphology, suggesting that integration facilitates phenotypic change of the avian neck. A large majority of the observed rate shifts (9 out of 12) are found within the ecological groups that exhibit significant head-neck or neck-forelimb integration, and this co-occurrence suggests that integration may be responsible for facilitating these shifts in the rate of neck morphological evolution. All but one of these particular rate shifts are found at the nodes and branches where a particular ecology is already established, rather than directly coinciding with a shift to a new dietary or foraging ecology (Fig. 3B, C). This suggests that there is a complex relationship between ecological transitions, trait correlation structure and transitions in evolutionary tempo, with the colonization of a new niche providing the opportunity for integration patterns and selective pressures to align.

Rate shifts are most frequently found in aquatic feeding and arboreal gleaning birds, both of which are niches that require extensive morphological adaptations across the entire body. The avian transition from land to water has involved a myriad of adaptations across the body to allow birds to effectively move through water [13, 46–48], a medium that is many times denser than air. Adaptations of the avian wing to a life aquatic have been well documented [41, 46, 47], and significant rate shifts of distal forelimb morphology have been observed at the base of Sphenisciformes + Procellariiformes [47]. Penguins display a highly modified neck that is able to provide strong, fast movements of the head to capture prey whilst keeping the head close to the body [49]. The latent evolutionary potential brought about by neck-forelimb integration in aquatic feeding birds may have synchronized the response to aquatic selective pressures on the neck [50] as the rate of forelimb morphological evolution increased at the base of Sphenisciformes + Procellariiformes [47], and subsequently manifested as a shift in the rate of neck evolution on the branch leading to penguins (Fig. 3A). By capitalizing on preexisting phenotypic covariance, penguins were able to evolve a neck morphology that allows them to participate in a novel and specialized foraging strategy, wing-propelled pursuit diving [46]. Similar to the land-to-water transition, the niche shift from ground foraging to arboreal gleaning necessitated the evolution of cranial, appendicular and axial morphological adaptations [51, 52]. In a terrestrial environment, the neck of birds must support the weight of the head during foraging, to allow for the successful procurement of food. During ground foraging, the head must be supported as it is lowered and raised to a relatively uniformly orientated surface; however, during arboreal gleaning, the head must be supported as it forages over a range of disparately orientated surfaces, from the near vertical orientation of tree bark to the inverted horizontality of the underside of branches and twigs [53, 54]. Strong integration between the head and neck within arboreal gleaning birds may have allowed for rapid bursts of neck evolution to accommodate a wider variety of foraging postures, better suited to support the head during arboreal gleaning.

We have shown that neck-forelimb integration is associated with a tradeoff between intervertebral stability and the relative length of the forelimb elements and that this pattern differs between the most cranial and caudal cervical vertebrae and the middle three vertebrae. Dorsoventral flexibility is highest in the middle cervical vertebrae of many bird species [55–58]. Indeed, middle vertebrae can act as a hinge for rapid strikes in some piscivorous taxa [59], and thus, the pattern recovered here matches previous functional data [55–58]. With the almost complete lack of co-occurrence of head-neck and neck-forelimb integration within a single ecological guild (Fig. 1), it appears that foraging capability (predominantly arboreal gleaning) and adaptations to the aquatic realm are two opposing forces that shape the evolution of the avian cervical spine. Phenotypic integration often occurs when traits share a developmental origin [60], and we suggest that avian neck-forelimb integration may occur due to the migration of neck muscle precursor cells during the formation of forelimb muscles during development, a pathway which is shared across Tetrapoda [61–63].

Mosaic evolution, whereby separate traits evolve at different rates or with different modes, is a central theme of avian macroevolution [7, 9–11]. A key aspect of evolutionary mosaicism in avians is the decoupling of the pectoral and pelvic locomotor modules. By emphasizing different combinations of these modules with the wing or tail, extant birds are able to successfully occupy a wide range of trophic niches [7, 9, 10]. Despite recent drives to increase our understanding of the tempo and mode of avian bauplan as a whole [41, 64], this work is among the first to quantify the role of the axial column in avian mosaicism (see [15, 27, 35]). Here, we find that the cervical column of extant birds is integrated with both the head and the forelimb, two major anatomical modules of the avian skeleton. The avian neck has previously been shown to be integrated with the pelvic module, with longer leg lengths correlated with longer necks [33]. Together, these findings suggest that the evolution of axial skeletal diversity in birds is shaped both by the strength and pattern of correlations between the vertebral column and other morpho-functional modules.

## Conclusions

This work expands on our current view of avian mosaicism—not only can birds emphasize different combinations of wing, tail and hindlimb modules to utilize a wide array of locomotor capabilities [7, 9, 10], but differential patterns of head-neck and neck-forelimb integration can allow birds to explore entirely new aquatic and arboreal niches. As foraging and dietary guild both significantly influence cervical morphological evolution, it is apparent that there is a complex web of selective pressures acting on the avian cervical column [15, 29, 33, 65]. We propose that it is the alignment of trophic ecology and integration between the neck, head and forelimb that influences the evolutionary variance of the avian cervical skeleton. This study shows that the avian neck acts to mediate between selective forces on both the cranial and pectoral modules by selectively integrating with either the head or the forelimb. This mediator role highlights the importance of the cervical skeleton as a keystone structure in avian mosaicism and macroevolution, a structure that mediates biomechanical function and evolutionary variance. Mosaicism is not unique to bird evolution [41] and may be ubiquitous across vertebrates [41, 50, 66, 67]; hence, it is of utmost importance that the cervical (and axial) skeleton be considered in future studies of vertebrate mosaicism and whole-organism modularity.

## Methods

### Resource availability

#### Lead contact

Further information and requests for resources should be directed to the lead contact, Ryan Marek (r.marek@ucl.ac.uk).

#### Materials availability

Computed tomography (CT) scan data is available at https://www.morphosource.org. For scans not available on -MorphoSource, please contact Ryan Marek (r.marek@ucl.ac.uk). A list of species analyzed can be found in Additional file 1: Table S1.

#### Digital code availability

All code is accessible on Dryad (https://doi.org/10.5061/dryad.m37pvmd7w).

### Experimental model and subject details

We analysed the morphology of the cervical vertebrae, head and forelimb in 112 species of extant birds, representing 93 families across 28 orders. Scan data for all species were downloaded from MorphoSource.

### Method details

#### Digitization and morphometric data collection

Scans were segmented in Avizo 8.1, and the resulting digital models were cleaned in MeshLab. For a valid comparison of cervical morphology across species with variable cervical counts, five vertebrae were chosen along the cervical column to be landmarked (C2 and vertebrae at 25%, 50%, 75% and 100%, i.e. last cervical vertebrae). We tested for the correspondence between the five vertebral positions used in this study and the five vertebral regions delineated by *Hox* expression limits and geometric morphometrics in a previous study [15]. Forty-five specimens from our dataset were also present in Marek et al. 2021, and correspondence between the vertebral regions of this current study and Marek et al. [15] were 100%, 91.11%, 82.22%, 71.11% and 100% for C2, C25%, C50%, C75% and the last cervical vertebrae, respectively. We used Stratovan Checkpoint to digitize landmarks on each vertebra, with 20 single points, 20 semi-landmark curves and 2 semilandmark patches for a total of 850 landmarks per vertebrae (Additional file 2: Fig. S1). Semilandmark patches were applied using a semi-automated template procedure [68, 69]. To reduce data dimensionality, we used the ‘lasec’ package in R to observe the minimum number of landmarks needed to adequately represent the shape variation across the data, giving a final total of 410 landmarks. Digital models of the skulls of all species were subjected to an α-shape fitting algorithm as part of an in-house modified version of the ‘alphavol’ MatLab package [15, 70] to produce head volumes, from which head mass was estimated by multiplying the volume by the weighted mean density of soft tissues within the skull (approximated to the density of water, 997 kg/m^3^). We acknowledge that this method may produce values that overestimate head mass; however, quantifying the amount of pneumatization and soft tissue within each skull was outside the scope of this study. This simplified metric of head morphology was used as head shape correlates poorly with ecology in birds [71–73], and head mass is one the largest constraints on overall neck morphology across vertebrates as the neck must resist the stress that this weight imposes upon it [25, 34, 74–76]. Forelimb morphology was assessed by digitally measuring the total length of each separate forelimb element in Geomagic Wrap. The forelimb elements included in this study are coracoid, scapula, humerus, radius, ulna and carpometacarpus. This simplified measure of forelimb morphology (element length and the relative proportions of these forelimb element lengths) adequately captures the main axes of variation in forelimb bone morphology [64] and has been repeatedly shown to correlate with flight style [47, 77–79]. Forelimb measurements were body size corrected by utilizing the following formula forelimb element/body mass^0.33, and head mass was taken as a percentage of total body mass. Body mass was calculated using scaling equations based on the length of the coracoid humeral articulation facet [80].

#### Phylogenetic and ecological framework

Phylogenetic trees were taken from www.birdtree.org and pruned to include only the 112 species within this study [81]. The effect of phylogeny on the morphological variation of cervical vertebrae was calculated using the *K*_mult_ statistic as part of the ‘geomorph’ R package. Dietary and foraging guilds were taken from the AVONET database [82]. Flesh feeders were designated as birds with diets consisting of at least 50% flesh (including piscivory), and birds were deemed aquatic if their predominant method of food acquisition involved a full-body submersion underwater. Variations existed between the number of species assigned to each ecological category. The number of species assigned to each dietary guild was as follows: aquatic animals *n* = 23, carrion *n* = 3, fruit *n* = 21, generalist *n* = 14, invertebrates *n* = 29, nectar *n* = 6, seeds *n* = 7, terrestrial plants *n* = 5, and vertebrates *n* = 4. The number of species assigned to each foraging guild was as follows: aerial sallying *n* = 12, aerial screening *n* = 11, aquatic dive *n* = 6, aquatic plunge *n* = 4, aquatic surface *n* = 1, arboreal gleaning *n* = 32, bark gleaning *n* = 6, foraging generalist *n* = 2 and ground foraging *n* = 38.

### Quantification and statistical analysis

#### Morphological analysis

After the manual placement of landmarks, semi-landmark curves were resampled to produce a consistent number across all vertebrae and then were slid to minimize bending energy. The data was then subjected to a Procrustes superimposition. The degree of neck-forelimb and head-neck integration was tested using a phylogenetic two-block partial least-squares analysis in the R package ‘geomorph’, with all forelimb elements being combined into a single matrix. To assess the integration between the entire neck and other skeletal elements, all five vertebral regions per species were pooled together. Integration between individual vertebral regions and other skeletal elements was also assessed. These tests were repeated for each individual dietary and foraging group, as well as for aquatic vs none-aquatic and for flesh versus none-flesh feeders. The ‘compare.pls’ function in the R package ‘geomorph’ was then used to compare for significant differences in effect sizes across the tests of integration of different ecological groupings. Phylogenetic MANOVAs were performed using the ‘procD.pgls’ function in the R package ‘geomorph’ to assess the relationship between vertebral morphology and ecological parameters, as well as the effect of body mass on vertebral shape variation. Due to the constraints imposed by the available data, there are discrepancies in the number of species assigned to each dietary and foraging guilds (see above), and we acknowledge that lack of significance in some of the above tests (see the ‘Results’ section) could be an issue of low power.

### Evolutionary rates

Rates of vertebral morphological evolution were calculated using a Markov chain Monte Carlo (MCMC) algorithm in BayesTraitsV3 (https://www.evolution.rdg.ac.uk) on the principal component (PC) scores of the total pooled dataset (all five vertebral regions pooled together per species) and for individual cervical regions. Separate models of evolution (Brownian motion, kappa, lambda, delta, Ornstein-Ulenbeck) were first tested to see which model best fit the data. Markov chains were run for 1,000,000,000 iterations with a burn-in of 500,000,000 iterations, and we confirmed convergence using the Gelman-Rubin test statistic^63^. Across the total pooled dataset and across all individual cervical regions, a lambda model with variable rates was deemed to best fit the data. Rates of evolution were then calculated using this model for the total dataset as well as for each cervical region. Differences in rate between vertebral regions were calculated using the compare.multi.evol.rates function in ‘geomorph’, and differences in rate across ecological groups were calculated using the ‘mvgls’ function in ‘mvMORPH’. Significant rate shifts at the branches and nodes were calculated using a modified version of the ‘plotShifts’ function in the ‘btrtools’ and ‘BTprocessR’ packages. The evolution of ecological traits (dietary and foraging guild) was mapped onto the phylogeny using the ‘make.simmap’ function in the ‘phytools’ package [83].

### Supplementary Information

**Additional file 1: Table S1. **Specimens studied with associated metadata (including forelimb proportion data, forelimb element length is adjusted for body mass). See associated Excel spreadsheet. **Table S2.** Phylogenetic signal (Kmult) of studied vertebral regions. **Table S3.** Phylogenetic MANOVA results. **Table S4.** Two-block partial least-squares (2BPLS) results for whole neck integration. **Table S5.** 2BPLS results for all groupings (diet, foraging, flesh feeding, aquatic dwelling). See associated Excel spreadsheet. **Table S6.** GIC and log-likelihood values for equal and multi-rates models of cervical evolution across the neck. **Table S7.** Average evolutionary rates per ecological group, averaged across all vertebral regions. **Table S8.** Evolutionary rates per dietary group across all vertebral regions. **Table S9.** Evolutionary rates per vertebral region.

**Additional file 2: Fig. S1.** Landmarks scheme used for cervical vertebrae. Red landmarks are fixed (invariant and variant), gold landmarks are landmarks that form curves, blue landmarks are landmarks that form patches.

## Data Availability

The dataset supporting the conclusions of this article is available in the Dryad repository (https://doi.org/10.5061/dryad.m37pvmd7w).

## Abbreviations

C2: Second cervical vertebrae
C25%: Vertebrae at 25% along the cervical column
C50%: Vertebrae at 50% along the cervical column
C75%: Vertebrae at 75% along the cervical column
*K*_mult_: Multivariate Blomberg's K-statistic
MANOVA: Multivariate analysis of variance
2BPLS: Two-block partial least squares analysis
PLS: Partial least squares
CT: Computed tomography
MCMC: Markov chain Monte Carlo
PC: Principal component
