# Supplementary material for: The neck as a keystone structure in avian macroevolution and mosaicism
Source: BMC Biol. 2023 Oct 13;21:216. doi: 10.1186/s12915-023-01715-x (PMC10576348; doi:10.1186/s12915-023-01715-x)
Supplement: Supplementary file 1 — Additional file 1: Table S1. Specimens studied with associated metadata (including forelimb proportion data, forelimb element length is adjusted for body mass). See associated Excel spreadsheet. Table S2. Phylogenetic signal (Kmult) of studied vertebral regions. Table S3. Phylogenetic MANOVA results. Table S4. Two-block partial least-squares (2BPLS) results for whole neck integration. Table S5. 2BPLS results for all groupings (diet, foraging, flesh feeding, aquatic dwelling). See associated Excel spreadsheet. Table S6. GIC and log-likelihood values for equal and multi-rates models of cervical evolution across the neck. Table S7. Average evolutionary rates per ecological group, averaged across all vertebral regions. Table S8. Evolutionary rates per dietary group across all vertebral regions. Table S9. Evolutionary rates per vertebral region. [file 12915_2023_1715_MOESM1_ESM.pdf]

Additional File 1 for Marek & Felice ‘The neck as a keystone structure in avian macroevolution and mosaicism’

Table S1. Specimens studied with associated metadata (including forelimb proportion data, forelimb element length is adjusted for body mass).

Species	Source	Diet	Foraging	Flesh	Water	Body mass (kg)	Coracoid length	Scapula length	Humerus length	Radius length	Ulna length	Carpometacarpus length	Head mass (% of body mass)	Brachial index	Coracoid:humerus	Coracoid:scapula	Ulna:CMC	Proximal	Distal	Proximal:distal
**Acanthorhynchus tenuirostris**	MorphoSource (ID: 000125748)	Nectar	Foraging generalist	Non-flesh	Non-aquatic	0.022638711	0.053753004	0.05824714	0.051122844	0.058282046	0.066067808	0.03676395	6.852342432	0.773793548	1.051447845	0.922843668	1.797081359	0.0467322	0.0461566	1.012470589
**Agelaius phoeniceus**	MorphoSource (ID: 000073651)	Seeds	Ground foraging	Non-flesh	Non-aquatic	0.260386577	0.040015609	0.044813273	0.044274015	0.045604777	0.052365065	0.029115088	2.320793979	0.845487646	0.903817036	0.892941009	1.798554256	0.0828114	0.081517	1.015878896
**Aptenodytes patagonicus**	MorphoSource (ID: 000375475)	Aquatic animals	Aquatic dive	Flesh	Aquatic	1.627086813	0.058174936	0.081786843	0.050751899	0.036040635	0.036539314	0.030890513	3.714775789	1.388966965	1.146261249	0.711299437	1.182865224	0.2239481	0.12150158	1.84317027
**Ardeotis kori**	MorphoSource (ID: 000125660)	Generalist	Ground foraging	Non-flesh	Non-aquatic	6.201364568	0.049231246	0.065210301	0.117577985	0.1255412	0.132988008	0.058003952	2.57503803	0.884124715	0.418711427	0.754961181	2.292740471	0.4236872	0.5780162	0.733002293
**Atlapetes leucopterus**	MorphoSource (ID: 000371807)	Generalist	Arboreal gleaning	Non-flesh	Non-aquatic	0.013799065	0.06390918	0.067463572	0.068863874	0.068729475	0.075246819	0.041523047	2.423388831	0.915173231	0.928050899	0.947313911	1.812169954	0.0487185	0.04513285	1.079446567
**Baeopogon indicator**	MorphoSource (ID: 000096282)	Fruit	Arboreal gleaning	Non-flesh	Non-aquatic	0.072285013	0.052948523	0.057927	0.050519846	0.058388415	0.064521739	0.03619908	5.047451537	0.782989537	1.048073707	0.914056017	1.782413768	0.067823	0.0668623	1.014368336
**Bonasa umbellus**	MorphoSource (ID: 000108161)	Terrestrial plants	Ground foraging	Non-flesh	Non-aquatic	0.246505838	0.057755949	0.079297018	0.071367602	0.059265762	0.067798246	0.041370087	3.78264591	1.052646737	0.809274051	0.728349562	1.638822911	0.1312936	0.1061043	1.237401312
**Calyptomena viridis**	MorphoSource (ID: 000108710)	Fruit	Aerial sallying	Non-flesh	Non-aquatic	0.764601841	0.066346845	0.073927612	0.076127588	0.08844068	0.09802956	0.051612631	1.564161811	0.776577876	0.871521698	0.897456892	1.899332738	0.1980592	0.2179023	0.908935794
**Caracara cheriway**	MorphoSource (ID: 000125586)	Carrion	Ground foraging	Flesh	Non-aquatic	1.846087544	0.043868775	0.051968147	0.087453943	0.087298581	0.092825153	0.049102919	3.092794715	0.942136266	0.501621463	0.844147373	1.890420256	0.2243903	0.2806263	0.799605383
**Cathartes burrovianus**	MorphoSource (ID: 000110050)	Carrion	Ground foraging	Flesh	Non-aquatic	2.236687199	0.043454737	0.045324585	0.10947194	0.121458124	0.128179014	0.060390253	2.144010259	0.854055098	0.396948629	0.958745378	2.122511604	0.2585745	0.4043615	0.639463698
**Charadrius hiaticula**	MorphoSource (ID: 000052994)	Aquatic animals	Ground foraging	Flesh	Aquatic	0.082141237	0.037048913	0.06421518	0.071850681	0.074144593	0.077452117	0.044871486	5.564630102	0.927678727	0.515637601	0.576949449	1.726087642	0.0758821	0.0861187	0.88113383
**Chloropsis hardwickii**	MorphoSource (ID: 000078331)	Generalist	Arboreal gleaning	Non-flesh	Non-aquatic	0.041275152	0.058738927	0.062862233	0.0627208	0.063559089	0.071966888	0.039476315	8.75103016	0.871523026	0.936514313	0.934407265	1.823039657	0.0643805	0.0611253	1.053254544
**Cisticola chubbi**	MorphoSource (ID: 000072802)	Invertebrates	Arboreal gleaning	Non-flesh	Non-aquatic	0.041612921	0.066648579	0.076357522	0.07582215	0.07141011	0.077780899	0.040949277	1.908734068	0.974817094	0.879012002	0.872848905	1.899444964	0.0766388	0.0665916	1.150877889
**Conostoma oemodium**	MorphoSource (ID: 000097883)	Generalist	Arboreal gleaning	Non-flesh	Non-aquatic	0.055554968	0.061428249	0.059853226	0.072996158	0.070693322	0.081402858	0.043631367	2.060150588	0.896727208	0.841527159	1.026314762	1.865695793	0.0748482	0.0754068	0.99259218
**Coracina novaehollandiae**	MorphoSource (ID: 000097881)	Invertebrates	Foraging generalist	Non-flesh	Non-aquatic	0.110326713	0.057793501	0.061957193	0.081448537	0.093751888	0.103524777	0.059834884	1.517485616	0.786754048	0.709570773	0.932797279	1.730174269	0.09721	0.1242242	0.782536736
**Corvus albus**	MorphoSource (ID: 000375493)	Generalist	Ground foraging	Non-flesh	Non-aquatic	1.780153699	0.053404623	0.061763738	0.084596588	0.100866415	0.109226275	0.064408854	1.760269915	0.774507674	0.631285779	0.864659823	1.695827012	0.2416408	0.3320441	0.727737069
**Crotophaga ani**	MorphoSource (ID:	Generalist	Arboreal	Non-	Non-aquatic	0.029096241	0.076643123	0.095571592	0.113517794	0.08744002	0.097065395	0.058880393	3.379240638	1.16949809	0.675163959	0.801944613	1.64851812	0.0889254	0.0757463	1.173990017

	000091735)		gleaning	flesh																
**Crypturellus tataupa**	MorphoSource (ID: 000109590)	Generalist	Ground foraging	Non-flesh	Non-aquatic	0.264289501	0.038204814	0.058974161	0.059855956	0.056282855	0.060365579	0.043280721	1.997428948	0.991557722	0.638279233	0.64782293	1.394745237	0.1012238	0.1030894	0.981903086
**Nectarinia oritis**	MorphoSource (ID: 000073432)	Nectar	Arboreal gleaning	Non-flesh	Non-aquatic	0.011223335	0.059213086	0.062590141	0.060012579	0.061842569	0.069489014	0.038264784	13.67935645	0.863626866	0.986677909	0.946044936	1.816004324	0.0413211	0.038544	1.072050125
**Emberiza cabanisi**	MorphoSource (ID: 000057347)	Invertebrates	Ground foraging	Non-flesh	Non-aquatic	0.14826702	0.063854281	0.054117743	0.071186441	0.071062721	0.080389424	0.043156018	8.830021673	0.885519983	0.897000609	1.179913967	1.862762755	0.1007559	0.1036587	0.971996562
**Eubucco bourcierii**	MorphoSource (ID: 000086173)	Fruit	Arboreal gleaning	Non-flesh	Non-aquatic	0.035117053	0.056881669	0.056057564	0.060582743	0.063459411	0.070203845	0.030954823	11.98090284	0.862954774	0.938908772	1.01470105	2.267945291	0.0574613	0.0545128	1.054088214
**Euneornis campestris**	MorphoSource (ID: 000095946)	Nectar	Arboreal gleaning	Non-flesh	Non-aquatic	0.023261466	0.057502644	0.059020314	0.059884836	0.066406978	0.074943224	0.040422541	9.345876997	0.79906939	0.960220446	0.974285631	1.853995858	0.0509927	0.0525435	0.970485407
**Forpus passerinus**	MorphoSource (ID: 000064737)	Seeds	Ground foraging	Non-flesh	Non-aquatic	0.042617026	0.045994784	0.047034757	0.052443408	0.056856564	0.062221588	0.038665115	9.623569228	0.842849078	0.877036517	0.977889272	1.609243574	0.0513505	0.0556818	0.922213362
**Foudia madagascariensis**	MorphoSource (ID: 000106437)	Seeds	Arboreal gleaning	Non-flesh	Non-aquatic	0.014005181	0.063644697	0.072609188	0.065741647	0.067708534	0.075335182	0.041004019	15.5608271	0.872655313	0.968103175	0.876537786	1.837263351	0.0493875	0.0449993	1.097517072
**Fratercula arctica**	MorphoSource (ID: 000086186)	Aquatic animals	Aquatic dive	Flesh	Aquatic	0.370061447	0.041911497	0.068802965	0.07412969	0.055293135	0.058699486	0.038468525	4.664942847	1.262867794	0.565380719	0.609152492	1.525909456	0.13314844	0.10982205	1.212401699
**Fregata aquila**	MorphoSource (ID: 000092063)	Aquatic animals	Aerial screaning	Flesh	Aquatic	1.431154052	0.057760336	0.056202917	0.15126792	0.199071259	0.202155268	0.092299141	5.035534148	0.748275923	0.38184128	1.02771064	2.190218317	0.298539	0.5555028	0.537421234
**Basilornis mirandus**	MorphoSource (ID: 000096285)	Fruit	Arboreal gleaning	Non-flesh	Non-aquatic	0.099890093	0.062088208	0.06800631	0.067725494	0.070661767	0.079975544	0.045892828	9.851476462	0.846827549	0.916762721	0.912977162	1.742658881	0.0924938	0.0918907	1.006563232
**Grus leucogeranus**	MorphoSource (ID: 000109469)	Generalist	Ground foraging	Flesh	Non-aquatic	2.733135312	0.059274652	0.081032763	0.16323772	0.177121498	0.183563629	0.080444637	4.993323946	0.889270497	0.363118598	0.731489955	2.281862848	0.422981575	0.614701873	0.688108486
**Lophozosterops goodfellowi**	MorphoSource (ID: 000159488)	Fruit	Arboreal gleaning	Non-flesh	Non-aquatic	0.018259415	0.063450232	0.068489211	0.0646284	0.068779849	0.074703651	0.04165561	12.07135059	0.865130404	0.981770126	0.926426684	1.793363499	0.052456962	0.04940704	1.061730515
**Heterocercus flavivertex**	MorphoSource (ID: 000052923)	Fruit	Aerial sallying	Non-flesh	Non-aquatic	0.025374057	0.061867533	0.068936314	0.07026823	0.085803482	0.092398219	0.046612379	12.38105913	0.760493343	0.880448147	0.897459251	1.982267798	0.059813668	0.066876291	0.894392723
**Heterophasia capistrata**	MorphoSource (ID: 000159441)	Generalist	Arboreal gleaning	Non-flesh	Non-aquatic	0.036582791	0.060896721	0.066681734	0.063085268	0.065549973	0.07389188	0.040854843	11.05838808	0.853751024	0.965308105	0.913244409	1.808644299	0.063995498	0.060515848	1.057499814
**Jacamerops aureus**	MorphoSource (ID: 000116074)	Invertebrates	Aerial sallying	Non-flesh	Non-aquatic	0.08440218	0.053396849	0.066323806	0.076584069	0.089844798	0.09706832	0.040894912	14.87027231	0.788970793	0.697231801	0.805093249	2.373603837	0.086821535	0.100754798	0.861711171
**Klais guimeti**	MorphoSource (ID: 000100075)	Nectar	Aerial screaning	Non-flesh	Non-aquatic	0.004859027	0.037102235	0.051997653	0.027479786	0.025360251	0.028975213	0.033088675	9.087127937	0.948389414	1.350164645	0.713536729	0.8756837	0.020099447	0.015072755	1.333495237
**Lanius excubitor**	MorphoSource (ID: 000072779)	Vertebrates	Aerial sallying	Flesh	Non-aquatic	0.042740102	0.065273127	0.072728565	0.075063442	0.084182912	0.091196968	0.053021683	2.086174245	0.823091421	0.869572791	0.897489546	1.71999384	0.075281475	0.080700236	0.93285322
**Lipaugus vociferans**	MorphoSource (ID: 000073648)	Fruit	Aerial sallying	Non-flesh	Non-aquatic	0.065893563	0.066801071	0.077604079	0.077556209	0.091982981	0.099528976	0.054156378	1.360263217	0.779232464	0.8613246	0.860793295	1.837807096	0.090468119	0.100130727	0.903500071

**Micrastur ruficollis**	MorphoSource (ID: 000093346)	Vertebrates	Aerial sallying	Flesh	Non-aquatic	0.101728773	0.069189455	0.08398278	0.111601051	0.112429686	0.122185021	0.059537821	1.827532118	0.913377517	0.619971357	0.82385288	2.052225262	0.12454624	0.13836589	0.900122422
**Neodrepanis hypoxantha**	MorphoSource (ID: 000042668)	Nectar	Arboreal gleaning	Non-flesh	Non-aquatic	0.007101578	0.060221403	0.066374482	0.06391465	0.072859283	0.080772874	0.042524021	16.8820648	0.791288545	0.94221595	0.907297525	1.899464657	0.037227691	0.038330907	0.97121863
**Opisthocomus hoazin**	MorphoSource (ID: 000126143)	Terrestrial plants	Arboreal gleaning	Non-flesh	Non-aquatic	0.457190352	0.061846519	0.078396689	0.088011243	0.083208217	0.089448128	0.04662042	4.220395272	0.983936106	0.702711566	0.788891976	1.918646972	0.176299582	0.169365382	1.040942251
**Pandion haliaetus**	MorphoSource (ID: 000093616)	Aquatic animals	Aerial screaning	Flesh	Aquatic	1.654270045	0.041163664	0.056393911	0.116957368	0.144590406	0.14960261	0.0715235	2.687392493	0.78178695	0.351954433	0.729931009	2.091656746	0.253278115	0.431802038	0.586560722
**Passerina cyanea**	MorphoSource (ID: 000114360)	Seeds	Ground foraging	Non-flesh	Non-aquatic	0.009845551	0.069845946	0.078992639	0.071584356	0.076668932	0.08458115	0.044724245	2.018312637	0.846339348	0.975715232	0.884208289	1.891170016	0.047976217	0.0448314	1.070147642
**Pelecanus occidentalis**	MorphoSource (ID: 000092472)	Aquatic animals	Aquatic plunge	Flesh	Aquatic	5.038209735	0.056239383	0.051790455	0.14409902	0.170202368	0.174708159	0.067828007	5.219606087	0.824798454	0.390282897	1.085902459	2.575752498	0.429906208	0.703762592	0.610868229
**Phaethon lepturus**	MorphoSource (ID: 000092495)	Aquatic animals	Aquatic plunge	Flesh	Aquatic	0.419743229	0.05922327	0.076788532	0.11234152	0.11631795	0.120869043	0.060383705	9.622232405	0.929448247	0.527171699	0.771251489	2.001683105	0.18648959	0.223447132	0.834602746
**Phainopepla nitens**	MorphoSource (ID: 000356971)	Fruit	Arboreal gleaning	Non-flesh	Non-aquatic	0.041401111	0.05082592	0.060948138	0.06532289	0.071808788	0.082129404	0.043401647	6.655422846	0.795365439	0.778072131	0.833920801	1.892310768	0.061919157	0.068996765	0.897421162
**Philepitta castanea**	MorphoSource (ID: 000042658)	Fruit	Arboreal gleaning	Non-flesh	Non-aquatic	0.026210622	0.06569326	0.074208733	0.068214696	0.079454158	0.088180563	0.048189801	14.67160528	0.773579722	0.963036768	0.885249719	1.829859467	0.062575514	0.064893068	0.964286571
**Pogoniulus bilineatus**	MorphoSource (ID: 000110058)	Fruit	Arboreal gleaning	Non-flesh	Non-aquatic	0.016361373	0.06065879	0.069166743	0.063803019	0.066802818	0.074494815	0.03205661	11.46325556	0.856475969	0.950719749	0.876993583	2.323851921	0.049834478	0.044616448	1.116953057
**Psalidoprocne pristoptera**	MorphoSource (ID: 000073409)	Invertebrates	Aerial screaning	Non-flesh	Non-aquatic	0.026158734	0.05091491	0.063649462	0.052841001	0.073725934	0.080291021	0.044838251	5.621051844	0.658118436	0.963549315	0.799926799	1.790681376	0.050301729	0.05975173	0.841845567
**Megalaima asiatica**	MorphoSource (ID: 000072684)	Fruit	Arboreal gleaning	Non-flesh	Non-aquatic	0.068689796	0.064409382	0.072495515	0.069524339	0.075900622	0.085532153	0.035563188	14.44228922	0.812844483	0.926429259	0.888460225	2.405075553	0.085299334	0.081401379	1.047885614
**Pterocles quadricinctus**	MorphoSource (ID: 000093681)	Seeds	Ground foraging	Non-flesh	Non-aquatic	0.086517589	0.044090852	0.066387876	0.069417747	0.072266095	0.077965313	0.04693374	2.713870124	0.890367065	0.63515245	0.664140118	1.661178365	0.080217128	0.087917353	0.912415186
**Ptilonorhynchus violaceus**	MorphoSource (ID: 000092979)	Generalist	Arboreal gleaning	Non-flesh	Non-aquatic	0.201184989	0.059587112	0.069099944	0.073333359	0.08031841	0.090371165	0.047756875	8.685697222	0.811468561	0.812551239	0.862332282	1.892317406	0.119009546	0.128686068	0.924805209
**Pycnonotus cafer**	MorphoSource (ID: 000092706)	Generalist	Arboreal gleaning	Non-flesh	Non-aquatic	0.072522961	0.044693268	0.052525106	0.049829221	0.050409803	0.058228748	0.029762739	4.998007459	0.85574949	0.896928887	0.850893437	1.956431072	0.061860703	0.058223333	1.06247272
**Sarothrura pulchra**	MorphoSource (ID: 000072793)	Aquatic animals	Ground foraging	Flesh	Aquatic	0.023169342	0.052307095	0.08864323	0.089431084	0.077231881	0.083995461	0.053559752	15.25113229	1.064713291	0.584887191	0.590085617	1.568257087	0.066507244	0.062005427	1.072603596
**Scissirostrum dubium**	MorphoSource (ID: 000086185)	Generalist	Arboreal gleaning	Non-flesh	Non-aquatic	0.10131196	0.052823744	0.059159102	0.051039002	0.053857208	0.060588667	0.036306676	5.186238624	0.842385293	1.034968201	0.892909834	1.668802371	0.076579733	0.070816214	1.081386997
**Spheniscus mendiculus**	MorphoSource (ID: 000097861)	Aquatic animals	Aquatic dive	Flesh	Aquatic	1.749297414	0.045915279	0.064159698	0.04292941	0.031791744	0.032784989	0.025849136	3.107101832	1.309422751	1.069552976	0.715640499	1.268320495	0.184013163	0.108752111	1.692042217
**Sula dactylatra**	MorphoSource (ID: 000093058)	Aquatic animals	Aquatic plunge	Flesh	Aquatic	1.213000579	0.056102131	0.0736192	0.162302741	0.165713315	0.171179687	0.07243484	7.277775998	0.94814253	0.345663483	0.762058417	2.363223095	0.311238121	0.436260023	0.713423428

**Surnia ulula**	MorphoSource (ID: 000157600)	Vertebrates	Aerial sallying	Flesh	Non-aquatic	0.110592079	0.073569653	0.087045122	0.138437019	0.148519691	0.155812796	0.079551293	2.558371292	0.88848299	0.53143049	0.845189845	1.958645683	0.144602346	0.185621676	0.779016487
**Topaza pyra**	MorphoSource (ID: 000107939)	Nectar	Aerial screaning	Non-flesh	Non-aquatic	0.024837009	0.032136181	0.046775626	0.023832669	0.022674812	0.026776628	0.029777988	5.033049672	0.890054899	1.348408836	0.687028349	0.899208787	0.030348781	0.023402879	1.296796903
**Tyranneutes stolzmanni**	MorphoSource (ID: 000052922)	Fruit	Aerial sallying	Non-flesh	Non-aquatic	0.011382884	0.052406557	0.059114344	0.051604179	0.059111545	0.064985688	0.033782845	11.37106378	0.79408529	1.015548694	0.886528607	1.92362985	0.037246374	0.036048782	1.033221428
**Tyto alba**	MorphoSource (ID: 000109995)	Vertebrates	Aerial sallying	Flesh	Non-aquatic	0.215686582	0.054710247	0.065339375	0.132211818	0.139627087	0.145858979	0.068811636	12.19045838	0.906435918	0.413807537	0.837324309	2.119684815	0.15205913	0.213564945	0.712004163
**Sterna hirundo**	MorphoSource (ID: 000109908)	Aquatic animals	Aquatic plunge	Flesh	Aquatic	0.188216169	0.039117344	0.05588921	0.095961178	0.106945631	0.111132738	0.058419903	4.318726198	0.863482529	0.407637179	0.699908694	1.902309517	0.110051705	0.159341613	0.690665187
**Strigops habroptila**	MorphoSource (ID: 000110026)	Terrestrial plants	Ground foraging	Non-flesh	Non-aquatic	0.377591713	0.055257743	0.058033797	0.096043783	0.085987785	0.095395765	0.053388004	17.011818	1.006792944	0.575339092	0.952164878	1.786838969	0.15179586	0.170240499	0.891655399
**Tauraco persa**	MorphoSource (ID: 000086199)	Fruit	Arboreal gleaning	Flesh	Aquatic	0.142114031	0.056405176	0.081043472	0.084744116	0.071649547	0.077610705	0.03817633	9.34870604	1.091912725	0.665594011	0.695986672	2.032953563	0.116707861	0.098452002	1.185429028
**Vultur gryphus**	MorphoSource (ID: 000110010)	Carrion	Ground foraging	Flesh	Non-aquatic	12.68856847	0.045777157	0.049635062	0.113869297	0.127620602	0.133410045	0.05778405	2.042571955	0.853528662	0.402014921	0.922274592	2.308769369	0.484013015	0.737334408	0.656436225
**Zavattariornis stresemanni**	MorphoSource (ID: 000086177)	Invertebrates	Ground foraging	Non-flesh	Non-aquatic	0.105550609	0.061127054	0.066631674	0.074989674	0.075170826	0.084264295	0.046123172	10.50825297	0.889934155	0.815139616	0.917387346	1.826940604	0.096538264	0.097876188	0.986330444
**Acanthisitta chloris**	MorphoSource (ID: 000109874)	Invertebrates	Bark gleaning	Non-flesh	Non-aquatic	0.008528506	0.045513749	0.050329696	0.050656796	0.059966887	0.065238076	0.035575953	7.479129404	0.776491264	0.898472716	0.904312036	1.833768907	0.0304107	0.0333751	0.911179292
**Anser fabalis**	MorphoSource (ID: 000108615)	Terrestrial plants	Ground foraging	Non-flesh	Aquatic	3.090205011	0.048350975	0.062983201	0.107258115	0.096394897	0.101485742	0.059555948	2.235330852	1.056878654	0.450790836	0.767680498	1.704040426	0.317198152	0.37356491	0.849111208
**Anseranas semipalmata**	MorphoSource (ID: 000083954)	Terrestrial plants	Ground foraging	Non-flesh	Aquatic	2.921563927	0.046938044	0.05988575	0.118157131	0.108954124	0.114596984	0.05286555	2.444668191	1.031066673	0.397251044	0.783793197	2.167706279	0.32047841	0.39374703	0.813919562
**Caprimulgus carolinensis**	MorphoSource (ID: 000065224)	Invertebrates	Aerial screaning	Non-flesh	Non-aquatic	0.192289036	0.042513376	0.053993086	0.07705227	0.088806443	0.092170573	0.048440542	12.16269918	0.835974736	0.551747217	0.787385551	1.902756831	0.10072829	0.13314708	0.756518956
**Aramus guarauna**	MorphoSource (ID: 000125551)	Aquatic animals	Ground foraging	Flesh	Non-aquatic	0.889805226	0.047001823	0.053211709	0.107637817	0.108693726	0.116344377	0.057209156	5.299755679	0.925165616	0.436666442	0.883298496	2.033667072	0.19999548	0.271579551	0.736415828
**Artamus leucorynchus**	MorphoSource (ID: 000368995)	Invertebrates	Aerial screaning	Non-flesh	Non-aquatic	0.031113196	0.067778849	0.074341218	0.074268869	0.089643405	0.098964248	0.050185041	4.654915554	0.750461607	0.912614534	0.911726374	1.971986996	0.068850449	0.075978858	0.906179045
**Barnardius zonarius**	MorphoSource (ID: 000108655)	Fruit	Arboreal gleaning	Non-flesh	Non-aquatic	0.231060657	0.044104948	0.050246242	0.056414769	0.059927581	0.065596125	0.043612023	8.537291574	0.860032033	0.781797904	0.877776057	1.50408353	0.092967869	0.104295349	0.891390363
**Bombycilla cedrorum**	MorphoSource (ID: 000114489)	Fruit	Arboreal gleaning	Non-flesh	Non-aquatic	0.040537095	0.059410057	0.075162226	0.060566287	0.064728886	0.073468153	0.041708991	3.212341684	0.824388319	0.980909668	0.790424387	1.761446441	0.067753929	0.062465049	1.084669428
**Bucorvus abyssinicus**	MorphoSource (ID: 000108667)	Invertebrates	Ground foraging	Non-flesh	Non-aquatic	1.661637699	0.066859623	0.082570775	0.123492475	0.15048768	0.16327191	0.062543918	4.619945073	0.756360817	0.541406451	0.809725024	2.610516184	0.322713345	0.444954146	0.725273262
**Buphagus erythrorhynchus**	MorphoSource (ID: 000072669)	Invertebrates	Bark gleaning	Non-flesh	Non-aquatic	0.102845546	0.049178673	0.051824218	0.056586297	0.057906014	0.066375784	0.039722034	14.76190228	0.852514171	0.869091568	0.94895157	1.671006686	0.074395665	0.077423929	0.960887234

**Capito niger**	MorphoSource (ID: 000108203)	Fruit	Arboreal gleaning	Non-flesh	Non-aquatic	0.057681765	0.067817542	0.069506333	0.062957606	0.067951349	0.076451105	0.032948181	2.553718667	0.823501589	1.077193787	0.975703062	2.320343707	0.078123828	0.069179189	1.129296673
**Cariama cristata**	MorphoSource (ID: 000375527)	Invertebrates	Ground foraging	Non-flesh	Non-aquatic	1.069593316	0.056414566	0.082414964	0.108666048	0.098867062	0.106584603	0.053228843	4.838660005	1.019528572	0.519155399	0.684518481	2.002384378	0.253051903	0.264487937	0.956761605
**Hirundo semirufa**	MorphoSource (ID: 000097699)	Invertebrates	Aerial screaning	Non-flesh	Non-aquatic	0.063283096	0.040978147	0.04987721	0.042001949	0.056251561	0.062205346	0.039310073	1.847009824	0.675214458	0.975624889	0.821580579	1.582427655	0.053433102	0.063451378	0.842110978
**Chlorospingus flavigularis**	MorphoSource (ID: 000097890)	Fruit	Arboreal gleaning	Non-flesh	Non-aquatic	0.011666513	0.065088833	0.070430118	0.067068753	0.068637077	0.077561847	0.042702212	7.729533238	0.864713206	0.970479254	0.924161919	1.81634259	0.046634104	0.043483563	1.072453607
**Chunga burmeisteri**	MorphoSource (ID: 000125536)	Invertebrates	Ground foraging	Non-flesh	Non-aquatic	0.68840681	0.058676787	0.083118946	0.099259608	0.089870611	0.094240428	0.050145954	6.104116692	1.053259303	0.591144661	0.705937578	1.879322681	0.213110899	0.207100652	1.029020898
**Cinclus mexicanus**	MorphoSource (ID: 000073645)	Aquatic animals	Ground foraging	Flesh	Aquatic	0.078803963	0.050723658	0.050624291	0.051864961	0.052173931	0.06099959	0.036730675	16.63118643	0.85025097	0.977994729	1.00196284	1.660726083	0.066245475	0.064814869	1.022072188
**Colinus virginianus**	MorphoSource (ID: 000109722)	Seeds	Ground foraging	Non-flesh	Non-aquatic	0.167302758	0.053940393	0.078715743	0.063926245	0.052261159	0.058273224	0.034109348	11.30564088	1.097008895	0.843791039	0.685255462	1.708423843	0.10896841	0.080178078	1.359079847
**Columba livia**	MorphoSource (ID: 000056052)	Seeds	Ground foraging	Non-flesh	Non-aquatic	0.220229098	0.060066488	0.074259014	0.080226431	0.085868182	0.093306048	0.057191592	12.42714257	0.859820259	0.748711959	0.808878073	1.631464432	0.130221017	0.143460827	0.907711322
**Coracias benghalensis**	MorphoSource (ID: 000108722)	Invertebrates	Aerial sallying	Non-flesh	Non-aquatic	0.209956432	0.048417046	0.058760127	0.12729758	0.144081166	0.152591063	0.066843739	17.01640796	0.834240081	0.380345374	0.823977896	2.282802612	0.140087285	0.217183148	0.645019129
**Dendropicos gabonensis**	MorphoSource (ID: 000045387)	Invertebrates	Bark gleaning	Non-flesh	Non-aquatic	0.150917932	0.043221504	0.035590927	0.05194179	0.058136733	0.063743443	0.029539067	10.01751071	0.814856995	0.832114262	1.214396718	2.157936868	0.070055171	0.081127025	0.863524467
**Dendroplex picus**	MorphoSource (ID: 000095985)	Invertebrates	Bark gleaning	Non-flesh	Non-aquatic	0.044328145	0.056012394	0.056468019	0.067355498	0.068945059	0.077973982	0.043833824	2.989626568	0.86382016	0.831593494	0.991931275	1.77885419	0.06431032	0.068214284	0.942769113
**Deroptyus accipitrinus**	MorphoSource (ID: 000110101)	Fruit	Arboreal gleaning	Non-flesh	Non-aquatic	0.434775163	0.045595203	0.048675716	0.062981159	0.068888433	0.075517325	0.044216676	6.043397884	0.833996167	0.723949883	0.936713559	1.707892378	0.119460342	0.143291591	0.833687038
**Dicaeum australe**	MorphoSource (ID: 000114367)	Fruit	Arboreal gleaning	Non-flesh	Non-aquatic	0.016982032	0.057848005	0.06287632	0.058832791	0.060880721	0.067055741	0.036613145	5.279586094	0.877371413	0.983261289	0.920028484	1.831466289	0.0467842	0.042873944	1.091203552
**Dryolimnas cuvieri**	MorphoSource (ID: 000170696)	Aquatic animals	Ground foraging	Flesh	Non-aquatic	0.132070684	0.044071102	0.069008751	0.080306735	0.060918355	0.066914132	0.044372345	14.19239488	1.20014611	0.548784633	0.638630633	1.508014339	0.099149944	0.088289987	1.123003269
**Enicurus schistaceus**	MorphoSource (ID: 000091719)	Aquatic animals	Ground foraging	Flesh	Non-aquatic	0.044658286	0.054309992	0.060382384	0.062170833	0.068242458	0.077131492	0.041454274	2.919433585	0.806036953	0.873560633	0.899434372	1.860640288	0.063402324	0.066974605	0.946662156
**Eudromia elegans**	MorphoSource (ID: 000092845)	Generalist	Ground foraging	Non-flesh	Non-aquatic	0.512321839	0.045691456	0.066638042	0.081623216	0.080284889	0.087220492	0.048964173	6.424468273	0.935826145	0.559785052	0.685666252	1.781312475	0.155541044	0.1735985	0.895981498
**Eurystomus gularis**	MorphoSource (ID: 000042765)	Invertebrates	Aerial screaning	Non-flesh	Non-aquatic	0.195312655	0.047546178	0.056397702	0.077962468	0.088077775	0.09240828	0.045523118	12.18374713	0.84367405	0.609859834	0.843051693	2.029919831	0.106117957	0.131846041	0.804862673
**Gavia stellata**	MorphoSource (ID: 000092076)	Aquatic animals	Aquatic dive	Flesh	Aquatic	1.343637692	0.046582166	0.058168466	0.123479278	0.098930906	0.101251664	0.066264494	4.058566184	1.219528389	0.377246826	0.800814759	1.5279927	0.251597127	0.293727127	0.856567555
**Glareola pratincola**	MorphoSource (ID: 000093274)	Invertebrates	Aerial screaning	Non-flesh	Non-aquatic	0.134734834	0.035332224	0.049949692	0.077365094	0.087606185	0.091281372	0.051052845	15.03781865	0.847545262	0.456694639	0.707356187	1.787978157	0.08394106	0.11867074	0.707344203

**Haematopus ostralegus**	MorphoSource (ID: 000093286)	Aquatic animals	Ground foraging	Flesh	Aquatic	0.442506865	0.04593491	0.069129493	0.098963692	0.097251495	0.102477131	0.056590958	8.121805749	0.965714894	0.46415922	0.664476302	1.810839312	0.16354015	0.19585533	0.835004848
**Hemiprocne comata**	MorphoSource (ID: 000092876)	Invertebrates	Aerial sallying	Non-flesh	Non-aquatic	0.03668162	0.032439177	0.044755246	0.038784281	0.043773287	0.046551835	0.036099718	2.22002736	0.833141823	0.83640012	0.724812849	1.289534583	0.03896245	0.042471776	0.917372751
**Hymenops perspicillatus**	MorphoSource (ID: 000092905)	Invertebrates	Ground foraging	Non-flesh	Non-aquatic	0.051763068	0.044915781	0.052373548	0.05377668	0.065641823	0.072396126	0.034886381	2.355801244	0.742811575	0.835227838	0.857604308	2.075197376	0.056858233	0.065085269	0.873596036
**Irena cyanogastra**	MorphoSource (ID: 000057165)	Fruit	Arboreal gleaning	Non-flesh	Non-aquatic	0.086651503	0.062996942	0.071258597	0.069937432	0.069579948	0.079399659	0.043218643	2.040863619	0.88082786	0.900761447	0.884060934	1.83716226	0.091097593	0.08574633	1.06240807
**Ixobrychus minutus**	MorphoSource (ID: 000092113)	Aquatic animals	Ground foraging	Flesh	Aquatic	0.171859381	0.055934103	0.062436449	0.101897011	0.100420933	0.105581679	0.055082331	15.66380133	0.965101259	0.54892781	0.895856566	1.91679759	0.123184969	0.14601215	0.843662455
**Jacana jacana**	MorphoSource (ID: 000092126)	Aquatic animals	Ground foraging	Flesh	Aquatic	0.157764145	0.039932246	0.064370988	0.080397609	0.084745373	0.091167993	0.046818934	14.04076573	0.881862224	0.496684504	0.620345406	1.947246226	0.100418012	0.121094925	0.829250375
**Phalacrocorax atriceps**	MorphoSource (ID: 000092511)	Aquatic animals	Aquatic dive	Flesh	Aquatic	1.434534922	0.066617913	0.08048157	0.125809066	0.132927737	0.137801742	0.057050105	4.716855823	0.912971518	0.529516001	0.827741225	2.415451156	0.307419783	0.369229652	0.832597765
**Megalaima chrysopogon**	MorphoSource (ID: 000092169)	Fruit	Arboreal gleaning	Non-flesh	Non-aquatic	0.128951527	0.052563066	0.059013362	0.056976063	0.061881165	0.069766097	0.028560634	12.96861882	0.81667264	0.922546475	0.890697702	2.442736321	0.085738507	0.081493817	1.052086037
**Menura novaehollandiae**	MorphoSource (ID: 000093330)	Invertebrates	Ground foraging	Non-flesh	Non-aquatic	0.56181882	0.057312815	0.074649644	0.080117698	0.075810319	0.084703652	0.049811904	6.215208668	0.945858848	0.715357729	0.76775738	1.70047007	0.17533427	0.173883942	1.008340782
**Mniotilta varia**	MorphoSource (ID: 000101435)	Invertebrates	Bark gleaning	Non-flesh	Non-aquatic	0.012190816	0.056055933	0.058759358	0.059490939	0.065077756	0.072520142	0.039832976	6.738703956	0.820336772	0.942260005	0.953991571	1.820605669	0.040710246	0.041440025	0.982389514
**Momotus momota**	MorphoSource (ID: 000092205)	Invertebrates	Aerial sallying	Non-flesh	Non-aquatic	0.08001906	0.06339276	0.073517961	0.086935439	0.101708707	0.107021735	0.046155551	2.599878079	0.812315731	0.729193531	0.862275812	2.318718617	0.097275503	0.1107643	0.878220717
**Monias benschi**	MorphoSource (ID: 000092221)	Invertebrates	Ground foraging	Non-flesh	Non-aquatic	0.060347584	0.067755833	0.073651902	0.077073699	0.071058763	0.076335915	0.04222062	2.6774477	1.009664953	0.879104471	0.919946823	1.808024522	0.086503225	0.075074273	1.152235267
**Myrmothera campanisona**	MorphoSource (ID: 000093373)	Invertebrates	Ground foraging	Non-flesh	Non-aquatic	0.036956478	0.063113633	0.069495798	0.072643261	0.067045926	0.07593849	0.037817971	3.518005693	0.956606594	0.868816077	0.908164733	2.008000101	0.069123664	0.06088945	1.135232195
**Numida meleagris**	MorphoSource (ID: 000093395)	Generalist	Ground foraging	Non-flesh	Non-aquatic	1.248953141	0.049844217	0.069424902	0.073324373	0.061051043	0.069596564	0.03846561	3.11652525	1.053563126	0.679776926	0.717958763	1.809319115	0.207253482	0.181985916	1.138843525
**Parus atricapillus**	MorphoSource (ID: 000093658)	Invertebrates	Arboreal gleaning	Non-flesh	Non-aquatic	0.001904536	0.050342191	0.05094694	0.051672547	0.057046012	0.06414793	0.0358147	2.059971563	0.805521654	0.974254108	0.98812983	1.791106146	0.019360344	0.019872568	0.974224569
**Pedionomus torquatus**	MorphoSource (ID: 000092430)	Invertebrates	Ground foraging	Non-flesh	Non-aquatic	0.062170217	0.045136561	0.069610058	0.087921146	0.093744057	0.09849089	0.048163199	2.849496247	0.892683029	0.513375481	0.648420095	2.044940764	0.081033923	0.096119898	0.843050447
**Pelagodroma marina**	MorphoSource (ID: 000092446)	Aquatic animals	Aerial screaning	Flesh	Aquatic	0.02671305	0.051671237	0.065624353	0.082976113	0.071775057	0.076119762	0.055481502	4.571915225	1.0900732	0.622724244	0.787378998	1.371984521	0.060595216	0.061534565	0.984734612
**Phalacrocorax harrisi**	MorphoSource (ID: 000110022)	Aquatic animals	Aquatic dive	Flesh	Aquatic	0.925642155	0.05947543	0.062262495	0.093390677	0.071824737	0.075877695	0.039715078	4.239344847	1.230805406	0.636845475	0.955236856	1.910551312	0.209712533	0.182699094	1.147857542
**Phoebastria irrorata**	MorphoSource (ID: 000092014)	Aquatic animals	Aquatic surface	Flesh	Aquatic	4.190631371	0.048407674	0.070443925	0.188909872	0.181858795	0.185570226	0.07024141	2.854175908	1.017996669	0.256247454	0.687180245	2.641892083	0.493817493	0.70226242	0.703180861

**Phoeniculus purpureus**	MorphoSource (ID: 000093633)	Invertebrates	Bark gleaning	Non-flesh	Non-aquatic	0.068296757	0.058480036	0.065971053	0.082714061	0.089082316	0.10002431	0.047282448	2.678542994	0.826939586	0.707014443	0.886449934	2.115463843	0.085441473	0.097494345	0.87637363

Table S2. Phylogenetic signal (K_{mult}) of studied vertebral regions.

Group	K_{mult}	Effect size	P
**All**	0.8459	7.7483	0.001
**C2**	0.771	6.2629	0.001
**C25%**	1.1564	6.799	0.001
**C50%**	0.7754	6.1272	0.001
**C75%**	0.8707	7.8927	0.001
**Last**	0.6937	7.7007	0.001

Table S3. Phylogenetic MANOVA results.

Vertebrae	Dependent variable	SS	MS	R^2	F	Z	P
**All**	Body mass	0.003471666	0.003471666	0.015962166	1.78431982	1.90451059	0.03
**All**	Diet	0.02018124	0.002522655	0.092790119	1.316864821	1.1102743	0.13
**All**	Foraging	0.022433283	0.00280416	0.103144652	1.480715256	2.14487298	0.014
**C2**	Body mass	0.000592618	0.000592618	0.012319566	1.372055411	1.04025489	0.157
**C2**	Diet	0.004076384	0.000509548	0.084741358	1.192061925	0.33388603	0.376
**C2**	Foraging	0.005225899	0.000653237	0.108637897	1.569185994	1.98975449	0.021
**C25%**	Body mass	0.000712957	0.000712957	0.016304204	1.823188043	1.52835232	0.074
**C25%**	Diet	0.003690035	0.000461254	0.084385316	1.186591871	0.24319304	0.391
**C25%**	Foraging	0.004399122	0.00054989	0.10060104	1.440115502	1.45134637	0.08
**C50%**	Body mass	0.000600605	0.000600605	0.018316015	2.052352608	1.92918207	0.029
**C50%**	Diet	0.003194898	0.000399362	0.097431446	1.389844415	0.86575954	0.195
**C50%**	Foraging	0.003137218	0.000392152	0.095672446	1.362097987	1.07204918	0.143
**C75%**	Body mass	0.000498443	0.000498443	0.013067709	1.456480881	1.11468044	0.132
**C75%**	Diet	0.004160787	0.000520098	0.109083512	1.576410622	1.91884657	0.024
**C75%**	Foraging	0.003811614	0.000476452	0.099929232	1.429430783	1.55043703	0.064
**Last**	Body mass	0.001067043	0.001067043	0.01949762	2.187387089	2.44433608	0.005
**Last**	Diet	0.005059136	0.000632392	0.092443469	1.311444105	1.24828378	0.104
**Last**	Foraging	0.005859429	0.000732429	0.107066881	1.543773053	2.47167332	0.007

Table S4. Two-block partial least-squares (2BPLS) results for whole neck integration.

Vertebrae	Dependent variable	Z	P	R (PLS)
**All**	Forelimb	2.7424	0.001	0.710198497
**All**	Forelimb (body mass corrected)	1.7008	0.033	0.568195804
**All**	Head mass	1.7901	0.024	0.579108491
**All**	Head mass (% of total body mass)	1.5916	0.049	0.556424289
**C2**	Forelimb	2.8782	0.001	0.592721833
**C2**	Forelimb (body mass corrected)	2.0902	0.003	0.496019439
**C2**	Head mass	2.3811	0.001	0.51617467
**C2**	Head mass (% of total body mass)	1.6541	0.048	0.433369586
**C25%**	Forelimb	1.9737	0.013	0.452578214
**C25%**	Forelimb (body mass corrected)	1.062	0.179	0.349481923
**C25%**	Head mass	1.7762	0.027	0.423513472
**C25%**	Head mass (% of total body mass)	2.1752	0.006	0.490760231
**C50%**	Forelimb	4.7814	0.001	0.689243869
**C50%**	Forelimb (body mass corrected)	3.6	0.001	0.552289802
**C50%**	Head mass	2.8379	0.002	0.476
**C50%**	Head mass (% of total body mass)	2.5605	0.003	0.438719216
**C75%**	Forelimb	4.6391	0.001	0.635993986
**C75%**	Forelimb (body mass corrected)	3.3917	0.001	0.522682979
**C75%**	Head mass	2.2838	0.01	0.429165792
**C75%**	Head mass (% of total body mass)	2.4875	0.007	0.441980193
**Last**	Forelimb	6.2587	0.001	0.766514241
**Last**	Forelimb (body mass corrected)	4.6609	0.001	0.582656011
**Last**	Head mass	3.8251	0.001	0.541632464
**Last**	Head mass (% of total body mass)	2.8623	0.002	0.495249459

Table S5. 2BPLS results for all groupings (diet, foraging, flesh feeding, aquatic dwelling).

Vertebrae	Dependent variable	Diet	Z	P	R (PLS)
**All**	Forelimb	Aquatic	2.380658713	0.008	0.777730929
**All**	Forelimb	Carrion	-0.35367384	0.5915	0.972854989

**All**	Forelimb	Fruit	-0.19548476	0.571	0.616913467
**All**	Forelimb	Generalist	0.809048062	0.22	0.774959608
**All**	Forelimb	Invertebrates	1.118030767	0.151	0.647960306
**All**	Forelimb	Nectar	1.571149267	0.083	0.937362148
**All**	Forelimb	Seeds	1.460411595	0.066	0.946137864
**All**	Forelimb	Terrestrial plants	0.815573732	0.2905	0.984563374
**All**	Forelimb	Vertebrates	-0.83891343	0.7695	0.782556005
**All**	Head mass	Aquatic	-0.38528702	0.653	0.555964399
**All**	Head mass	Carrion	-0.52951102	0.7535	0.968277497
**All**	Head mass	Fruit	2.063353083	0.019	0.790015108
**All**	Head mass	Generalist	1.762485485	0.044	0.847754698
**All**	Head mass	Invertebrates	0.738092283	0.241	0.617827559
**All**	Head mass	Nectar	1.059115723	0.165	0.907470322
**All**	Head mass	Seeds	-0.89991425	0.788	0.739353843
**All**	Head mass	Terrestrial plants	0.469618768	0.3085	0.967260961
**All**	Head mass	Vertebrates	0.759231017	0.2275	0.822674784
**C2**	Forelimb	Aquatic	1.699288697	0.044	0.645571629
**C2**	Forelimb	Carrion	-0.72496445	0.5915	0.995399811
**C2**	Forelimb	Fruit	0.531719246	0.298	0.682220214
**C2**	Forelimb	Generalist	0.687985707	0.256	0.698212461
**C2**	Forelimb	Invertebrates	1.321215738	0.113	0.639986032
**C2**	Forelimb	Nectar	0.740830636	0.23	0.903365388
**C2**	Forelimb	Seeds	1.011811388	0.174	0.901533691
**C2**	Forelimb	Terrestrial plants	1.192682731	0.1255	0.93477309
**C2**	Forelimb	Vertebrates	-0.32626427	0.6415	0.863893189
**C2**	Head mass	Aquatic	-1.06852219	0.848	0.360233211
**C2**	Head mass	Carrion	-0.95134113	0.7505	0.995018541
**C2**	Head mass	Fruit	1.208933322	0.121	0.73560746
**C2**	Head mass	Generalist	2.518981293	0.008	0.872726813
**C2**	Head mass	Invertebrates	0.360969991	0.364	0.534144834
**C2**	Head mass	Nectar	-0.25336864	0.592	0.81827115
**C2**	Head mass	Seeds	0.734935354	0.239	0.883984123
**C2**	Head mass	Terrestrial plants	1.545861531	0.0825	0.94498344
**C2**	Head mass	Vertebrates	0.201122688	0.4605	0.943942584
**C25%**	Forelimb	Aquatic	-0.00532348	0.487	0.4039273

**C25%**	Forelimb	Carrion	-0.93050285	0.7505	0.964730573
**C25%**	Forelimb	Fruit	2.024529441	0.016	0.809904113
**C25%**	Forelimb	Generalist	0.253712155	0.4	0.590461702
**C25%**	Forelimb	Invertebrates	0.443325486	0.327	0.563242553
**C25%**	Forelimb	Nectar	1.555122576	0.061	0.953411486
**C25%**	Forelimb	Seeds	2.064581631	0.012	0.947934898
**C25%**	Forelimb	Terrestrial plants	1.227859301	0.1485	0.908714683
**C25%**	Forelimb	Vertebrates	-0.00407704	0.4885	0.870433954
**C25%**	Head mass	Aquatic	-0.35293394	0.632	0.375076411
**C25%**	Head mass	Carrion	-0.88553993	0.5885	0.96614617
**C25%**	Head mass	Fruit	-0.06306305	0.523	0.614893328
**C25%**	Head mass	Generalist	2.242264335	0.012	0.80300922
**C25%**	Head mass	Invertebrates	0.767209327	0.25	0.597678793
**C25%**	Head mass	Nectar	-0.20934651	0.556	0.847627975
**C25%**	Head mass	Seeds	1.061987533	0.157	0.866697756
**C25%**	Head mass	Terrestrial plants	1.472713711	0.0845	0.943709608
**C25%**	Head mass	Vertebrates	-0.72638175	0.7335	0.829056272
**C50%**	Forelimb	Aquatic	1.383194619	0.083	0.565020686
**C50%**	Forelimb	Carrion	1.026911634	0.2505	0.998206162
**C50%**	Forelimb	Fruit	0.228179368	0.416	0.668099347
**C50%**	Forelimb	Generalist	0.957306361	0.179	0.730587374
**C50%**	Forelimb	Invertebrates	2.479359284	0.012	0.738135801
**C50%**	Forelimb	Nectar	1.613752768	0.048	0.978041225
**C50%**	Forelimb	Seeds	1.057788853	0.159	0.860281957
**C50%**	Forelimb	Terrestrial plants	1.675764037	0.0095	0.993886412
**C50%**	Forelimb	Vertebrates	1.391634272	0.0255	0.994651026
**C50%**	Head mass	Aquatic	0.399394998	0.351	0.464880913
**C50%**	Head mass	Carrion	0.785469355	0.2505	0.996972567
**C50%**	Head mass	Fruit	1.235734727	0.116	0.746603451
**C50%**	Head mass	Generalist	1.394855952	0.083	0.768528267
**C50%**	Head mass	Invertebrates	1.294450528	0.099	0.622319802
**C50%**	Head mass	Nectar	0.103537505	0.45	0.887130345
**C50%**	Head mass	Seeds	-1.78794862	0.966	0.621889464
**C50%**	Head mass	Terrestrial plants	1.337789204	0.1105	0.978651669
**C50%**	Head mass	Vertebrates	-1.59078283	0.8965	0.805238491

**C75%**	Forelimb	Aquatic	2.031428486	0.021	0.680731979
**C75%**	Forelimb	Carrion	-0.50058542	0.7535	0.952005966
**C75%**	Forelimb	Fruit	0.409722509	0.35	0.609854882
**C75%**	Forelimb	Generalist	0.885776199	0.203	0.673089734
**C75%**	Forelimb	Invertebrates	1.902100316	0.035	0.677676369
**C75%**	Forelimb	Nectar	1.334103999	0.091	0.936996851
**C75%**	Forelimb	Seeds	0.884808547	0.204	0.861945325
**C75%**	Forelimb	Terrestrial plants	-0.1790324	0.5715	0.836524653
**C75%**	Forelimb	Vertebrates	0.709593179	0.3235	0.891051903
**C75%**	Head mass	Aquatic	0.029524544	0.492	0.462396131
**C75%**	Head mass	Carrion	-0.62153618	0.7535	0.94349485
**C75%**	Head mass	Fruit	1.592398957	0.056	0.730083336
**C75%**	Head mass	Generalist	0.591514499	0.292	0.636104234
**C75%**	Head mass	Invertebrates	-0.24871595	0.592	0.464778565
**C75%**	Head mass	Nectar	0.369183725	0.378	0.874535907
**C75%**	Head mass	Seeds	1.146460406	0.134	0.886451949
**C75%**	Head mass	Terrestrial plants	-0.04356335	0.5315	0.849834005
**C75%**	Head mass	Vertebrates	-0.49202449	0.6365	0.782906148
**Last**	Forelimb	Aquatic	2.380658713	0.008	0.777730929
**Last**	Forelimb	Carrion	-0.35367384	0.5915	0.972854989
**Last**	Forelimb	Fruit	-0.19548476	0.571	0.616913467
**Last**	Forelimb	Generalist	0.809048062	0.22	0.774959608
**Last**	Forelimb	Invertebrates	1.118030767	0.151	0.647960306
**Last**	Forelimb	Nectar	1.571149267	0.083	0.937362148
**Last**	Forelimb	Seeds	1.460411595	0.066	0.946137864
**Last**	Forelimb	Terrestrial plants	0.815573732	0.2905	0.984563374
**Last**	Forelimb	Vertebrates	-0.83891343	0.7695	0.782556005
**Last**	Head mass	Aquatic	-0.38528702	0.653	0.555964399
**Last**	Head mass	Carrion	-0.52951102	0.7535	0.968277497
**Last**	Head mass	Fruit	2.063353083	0.019	0.790015108
**Last**	Head mass	Generalist	1.762485485	0.044	0.847754698
**Last**	Head mass	Invertebrates	0.738092283	0.241	0.617827559
**Last**	Head mass	Nectar	1.059115723	0.165	0.907470322
**Last**	Head mass	Seeds	-0.89991425	0.788	0.739353843
**Last**	Head mass	Terrestrial plants	0.469618768	0.3085	0.967260961

**Last**	Head mass	Vertebrates	0.759231017	0.2275	0.822674784
**All**	Forelimb	Aerial sallying	1.692885486	0.033	0.898902972
**All**	Forelimb	Aerial screening	2.112968827	0.015	0.957638713
**All**	Forelimb	Aquatic dive	0.976890937	0.19	0.890443196
**All**	Forelimb	Aquatic plunge	0.863704152	0.2355	0.929834548
**All**	Forelimb	Arboreal gleaning	-0.028318013	0.512	0.69586495
**All**	Forelimb	Bark gleaning	0.345158613	0.397	0.877764766
**All**	Forelimb	Foraging generalist	0.990049504	0.5005	1
**All**	Forelimb	Ground foraging	1.293000084	0.123	0.654564549
**All**	Head mass	Aerial sallying	0.445481463	0.355	0.818196635
**All**	Head mass	Aerial screening	-0.748078371	0.747	0.826578702
**All**	Head mass	Aquatic dive	-1.037179442	0.816	0.73218412
**All**	Head mass	Aquatic plunge	-1.587714878	0.9315	0.757441893
**All**	Head mass	Arboreal gleaning	1.869552573	0.032	0.807248503
**All**	Head mass	Bark gleaning	1.248997138	0.11	0.925958161
**All**	Head mass	Foraging generalist	-0.990049504	0.5005	1
**All**	Head mass	Ground foraging	1.286315805	0.115	0.660128608
**C2**	Forelimb	Aerial sallying	1.249715777	0.12	0.806992291
**C2**	Forelimb	Aerial screening	2.133547409	0.015	0.927080287
**C2**	Forelimb	Aquatic dive	0.153687649	0.452	0.683586759
**C2**	Forelimb	Aquatic plunge	0.91815476	0.2295	0.92183754
**C2**	Forelimb	Arboreal gleaning	0.306494387	0.381	0.595318302
**C2**	Forelimb	Bark gleaning	-0.085353622	0.546	0.751523012
**C2**	Forelimb	Foraging generalist	0.990049504	0.5005	1
**C2**	Forelimb	Ground foraging	0.721484493	0.245	0.484993018
**C2**	Head mass	Aerial sallying	1.437393693	0.078	0.813397274
**C2**	Head mass	Aerial screening	0.331786442	0.389	0.798430275
**C2**	Head mass	Aquatic dive	-1.368416516	0.899	0.488530966
**C2**	Head mass	Aquatic plunge	-1.990142486	0.9755	0.824105057
**C2**	Head mass	Arboreal gleaning	1.947616616	0.029	0.726122102
**C2**	Head mass	Bark gleaning	0.324326791	0.37	0.786022586
**C2**	Head mass	Foraging generalist	-0.990049504	0.5005	1
**C2**	Head mass	Ground foraging	1.386934208	0.104	0.581900374
**C25%**	Forelimb	Aerial sallying	1.517920158	0.065	0.831133964
**C25%**	Forelimb	Aerial screening	1.722866429	0.042	0.888437248

**C25%**	Forelimb	Aquatic dive	1.405485099	0.07	0.881230425
**C25%**	Forelimb	Aquatic plunge	0.920086801	0.1515	0.905225223
**C25%**	Forelimb	Arboreal gleaning	0.808776984	0.225	0.629582937
**C25%**	Forelimb	Bark gleaning	-1.740914399	0.961	0.528152583
**C25%**	Forelimb	Foraging generalist	0.990049504	0.5005	1
**C25%**	Forelimb	Ground foraging	1.01474541	0.173	0.46601881
**C25%**	Head mass	Aerial sallying	0.376026522	0.361	0.720463798
**C25%**	Head mass	Aerial screening	-0.238544167	0.572	0.668174998
**C25%**	Head mass	Aquatic dive	-1.615733559	0.95	0.557728211
**C25%**	Head mass	Aquatic plunge	-1.425716555	0.8865	0.555930741
**C25%**	Head mass	Arboreal gleaning	2.399361398	0.006	0.75223333
**C25%**	Head mass	Bark gleaning	0.400405356	0.346	0.727313747
**C25%**	Head mass	Foraging generalist	-0.990049504	0.5005	1
**C25%**	Head mass	Ground foraging	0.401202886	0.348	0.41546886
**C50%**	Forelimb	Aerial sallying	-0.48392651	0.686	0.703289328
**C50%**	Forelimb	Aerial screening	2.799705558	0.002	0.948449162
**C50%**	Forelimb	Aquatic dive	1.206216883	0.149	0.859345247
**C50%**	Forelimb	Aquatic plunge	0.49114258	0.3645	0.933478785
**C50%**	Forelimb	Arboreal gleaning	-0.001012341	0.501	0.596764161
**C50%**	Forelimb	Bark gleaning	-0.207442014	0.554	0.849380737
**C50%**	Forelimb	Foraging generalist	0.990049504	0.5005	1
**C50%**	Forelimb	Ground foraging	2.454219364	0.005	0.666894747
**C50%**	Head mass	Aerial sallying	0.312825275	0.398	0.763841595
**C50%**	Head mass	Aerial screening	-0.205878574	0.57	0.736766846
**C50%**	Head mass	Aquatic dive	-0.525309714	0.653	0.608061502
**C50%**	Head mass	Aquatic plunge	-1.286802277	0.8345	0.706598969
**C50%**	Head mass	Arboreal gleaning	1.76740392	0.039	0.712956822
**C50%**	Head mass	Bark gleaning	-1.246034479	0.879	0.779317907
**C50%**	Head mass	Foraging generalist	-0.990049504	0.5005	1
**C50%**	Head mass	Ground foraging	2.584698199	0.003	0.685508722
**C75%**	Forelimb	Aerial sallying	1.525526475	0.07	0.838783255
**C75%**	Forelimb	Aerial screening	2.19784872	0.015	0.892082374
**C75%**	Forelimb	Aquatic dive	0.895019328	0.202	0.905461199
**C75%**	Forelimb	Aquatic plunge	0.762052998	0.2745	0.937098254
**C75%**	Forelimb	Arboreal gleaning	1.297084519	0.112	0.637834347

**C75%**	Forelimb	Bark gleaning	1.251065072	0.117	0.918386211
**C75%**	Forelimb	Foraging generalist	0.990049504	0.5005	1
**C75%**	Forelimb	Ground foraging	1.616023241	0.05	0.563834453
**C75%**	Head mass	Aerial sallying	0.555765449	0.305	0.76719509
**C75%**	Head mass	Aerial screening	-0.763971878	0.772	0.624231289
**C75%**	Head mass	Aquatic dive	-0.874692986	0.822	0.750501745
**C75%**	Head mass	Aquatic plunge	-0.138423856	0.5325	0.803894526
**C75%**	Head mass	Arboreal gleaning	2.080549924	0.014	0.704794926
**C75%**	Head mass	Bark gleaning	1.742490277	0.034	0.95128162
**C75%**	Head mass	Foraging generalist	-0.990049504	0.5005	1
**C75%**	Head mass	Ground foraging	2.324715181	0.003	0.624417022
**Last**	Forelimb	Aerial sallying	1.04780167	0.156	0.795097409
**Last**	Forelimb	Aerial screening	0.964436145	0.182	0.840856497
**Last**	Forelimb	Aquatic dive	0.283123726	0.385	0.915626767
**Last**	Forelimb	Aquatic plunge	0.179830957	0.4755	0.906958206
**Last**	Forelimb	Arboreal gleaning	0.265647529	0.4	0.581249043
**Last**	Forelimb	Bark gleaning	0.461836109	0.376	0.871217417
**Last**	Forelimb	Foraging generalist	0.990049504	0.5005	1
**Last**	Forelimb	Ground foraging	2.405228414	0.006	0.665055753
**Last**	Head mass	Aerial sallying	0.482069708	0.335	0.742358142
**Last**	Head mass	Aerial screening	-0.938530057	0.803	0.686202114
**Last**	Head mass	Aquatic dive	-0.602276492	0.683	0.858808112
**Last**	Head mass	Aquatic plunge	-0.899951949	0.7635	0.854636907
**Last**	Head mass	Arboreal gleaning	1.88229622	0.026	0.705352289
**Last**	Head mass	Bark gleaning	-0.096051051	0.528	0.827379316
**Last**	Head mass	Foraging generalist	-0.990049504	0.5005	1
**Last**	Head mass	Ground foraging	0.800555047	0.212	0.52437068
**All**	Forelimb	Flesh	1.817632108	0.032	0.709140294
**All**	Forelimb	Non-flesh	1.509241226	0.068	0.625038717
**All**	Head mass	Flesh	-0.551707067	0.675	0.448153431
**All**	Head mass	Non-flesh	0.52113481	0.331	0.543667292
**C2**	Forelimb	Flesh	1.208451597	0.142	0.514220583
**C2**	Forelimb	Non-flesh	1.565020074	0.052	0.497801255
**C2**	Head mass	Flesh	-0.985155892	0.832	0.313282869
**C2**	Head mass	Non-flesh	1.168378439	0.134	0.462715335

**C25%**	Forelimb	Flesh	-0.265180082	0.598	0.32675704
**C25%**	Forelimb	Non-flesh	2.250378931	0.003	0.586354954
**C25%**	Head mass	Flesh	-0.113547851	0.526	0.338693338
**C25%**	Head mass	Non-flesh	0.646840131	0.286	0.413592477
**C50%**	Forelimb	Flesh	1.404395633	0.076	0.499457833
**C50%**	Forelimb	Non-flesh	2.899177405	0.002	0.583228639
**C50%**	Head mass	Flesh	-0.48069518	0.673	0.341058306
**C50%**	Head mass	Non-flesh	1.250798887	0.108	0.458332229
**C75%**	Forelimb	Flesh	1.910659546	0.021	0.598121974
**C75%**	Forelimb	Non-flesh	2.094583728	0.013	0.490240632
**C75%**	Head mass	Flesh	0.359481322	0.377	0.451325448
**C75%**	Head mass	Non-flesh	0.647631221	0.279	0.388458649
**Last**	Forelimb	Flesh	2.436024273	0.005	0.706408642
**Last**	Forelimb	Non-flesh	3.089646007	0.002	0.578682299
**Last**	Head mass	Flesh	-0.739565263	0.77	0.459699037
**Last**	Head mass	Non-flesh	2.568452081	0.005	0.529643735
**All**	Forelimb	Aquatic	1.908562777	0.032	0.738722346
**All**	Forelimb	Non-aquatic	1.962282886	0.02	0.637999192
**All**	Head mass	Aquatic	0.672700985	0.269	0.615857332
**All**	Head mass	Non-aquatic	0.920862648	0.218	0.55017389
**C2**	Forelimb	Aquatic	2.328453122	0.005	0.741497892
**C2**	Forelimb	Non-aquatic	2.159464373	0.008	0.553317989
**C2**	Head mass	Aquatic	1.941431618	0.021	0.675628347
**C2**	Head mass	Non-aquatic	1.762694839	0.031	0.492935287
**C25%**	Forelimb	Aquatic	0.444003145	0.334	0.448252591
**C25%**	Forelimb	Non-aquatic	2.227787518	0.003	0.565265801
**C25%**	Head mass	Aquatic	0.277571474	0.402	0.430491754
**C25%**	Head mass	Non-aquatic	1.616474736	0.05	0.485621598
**C50%**	Forelimb	Aquatic	1.198754212	0.106	0.541839321
**C50%**	Forelimb	Non-aquatic	2.926304814	0.002	0.537195819
**C50%**	Head mass	Aquatic	0.143648842	0.443	0.432780115
**C50%**	Head mass	Non-aquatic	1.492804429	0.075	0.450264853
**C75%**	Forelimb	Aquatic	2.315032542	0.011	0.692682326
**C75%**	Forelimb	Non-aquatic	1.829554878	0.034	0.44359345
**C75%**	Head mass	Aquatic	0.397567519	0.345	0.506243013

**C75%**	Head mass	Non-aquatic	0.870474501	0.193	0.383496098
**Last**	Forelimb	Aquatic	2.193574387	0.014	0.765995195
**Last**	Forelimb	Non-aquatic	3.094361412	0.002	0.565989793
**Last**	Head mass	Aquatic	0.646470873	0.266	0.634200679
**Last**	Head mass	Non-aquatic	2.67094735	0.004	0.514240179

Table S6. GIC and log-likelihood values for equal and multi-rates models of cervical evolution across the neck.

Model	GIC	logLik
**C2 equal rates diet**	-1583821	796918.4
**C2 multi-rates diet**	-1599521	804785.8
**C25% equal rates diet**	-1599312	804648.8
**C25% multi-rates diet**	-1614751	812368.5
**C50% equal rates diet**	-1643529	826709.4
**C50% multi-rates diet**	-1671206	840590.3
**C75% equal rates diet**	-1624524	817158.1
**C75% multi-rates diet**	-1642845	826362.4
**Last equal rates diet**	-1550408	780185.1
**Last multi-rates diet**	-1567778	788876.8
**C2 equal rates foraging**	-1583821	796918.4
**C2 multi-rates foraging**	-1603531	806754.5
**C25% equal rates foraging**	-1599312	804648.8
**C25% multi-rates foraging**	-1618821	814383.8
**C50% equal rates foraging**	-1643529	826709.4
**C50% multi-rates foraging**	-1661518	835709.7
**C75% equal rates foraging**	-1624524	817158.1
**C75% multi-rates foraging**	-1643723	826745.5
**Last equal rates foraging**	-1550408	780185.1
**Last multi-rates foraging**	-1573061	791508.3
**C2 equal rates flesh**	-1583821	796918.4
**C2 multi-rates flesh**	-1593901	801943.8
**C25% equal rates flesh**	-1599312	804648.8
**C25% multi-rates flesh**	-1607209	808562.5

**C50% equal rates flesh**	-1643529	826709.4
**C50% multi-rates flesh**	-1645612	827742.1
**C75% equal rates flesh**	-1624524	817158.1
**C75% multi-rates flesh**	-1639129	824468.3
**Last equal rates flesh**	-1550408	780185.1
**Last multi-rates flesh**	-1559618	784756.5
**C2 equal rates water**	-1583821	796918.4
**C2 multi-rates water**	-1586001	797995
**C25% equal rates water**	-1599312	804648.8
**C25% multi-rates water**	-1599461	804724.3
**C50% equal rates water**	-1643529	826709.4
**C50% multi-rates water**	-1643960	826924.6
**C75% equal rates water**	-1624524	817158.1
**C75% multi-rates water**	-1627022	818394.5
**Last equal rates water**	-1550408	780185.1
**Last multi-rates water**	-1550514	780238.6

Table S7. Average evolutionary rates per ecological group, averaged across all vertebral regions.

Ecology	Grouping	Average rate
Foraging generalist	Foraging	9.95E-16
Aquatic surface	Foraging	6.85E-13
Vertebrates	Diet	1.96E-12
Carrion	Diet	2.49E-12
Aerial screening	Foraging	2.17E-08
Flesh	Flesh	1.09E-07
Aerial sallying	Foraging	2.02E-07
Aquatic	Aquatic	2.51E-07
Aquatic animals	Diet	2.63E-07
Terrestrial plants	Diet	2.9E-07
Non-aquatic	Aquatic	3.05E-07
Bark gleaning	Foraging	3.23E-07

Seeds	Diet	3.4E-07
Aquatic dive	Foraging	3.64E-07
Nectar	Diet	3.67E-07
Aquatic plunge	Foraging	3.8E-07
Non-flesh	Flesh	4.76E-07
Generalist	Diet	5.17E-07
Invertebrates	Diet	5.29E-07
Fruit	Diet	5.47E-07
Ground foraging	Foraging	5.59E-07
Arboreal gleaning	Foraging	5.75E-07

Table S8. Evolutionary rates per dietary group across all vertebral regions.

Group	C2	C25%	C50%	C75%	Last
**Terrestrial plants**	2.45E-07	4.79E-07	1.20E-07	3.46E-07	2.60E-07
**Nectar**	4.28E-07	4.12E-07	2.72E-07	3.01E-07	4.20E-07
**Seeds**	3.28E-07	3.29E-07	3.56E-07	3.22E-07	3.63E-07
**Aquatic animals**	2.48E-07	3.52E-07	3.72E-07	1.21E-07	2.22E-07
**Generalist**	4.61E-07	5.22E-07	5.00E-07	5.72E-07	5.29E-07
**Fruit**	6.82E-07	1.89E-07	5.24E-07	4.13E-07	9.25E-07
**Carrion**	1.62E-12	3.33E-14	2.32E-14	6.50E-13	1.01E-11
**Invertebrates**	7.24E-07	5.96E-07	8.63E-08	3.93E-07	8.45E-07
**Vertebrates**	2.14E-13	3.31E-15	9.60E-12	1.24E-15	2.70E-15
**Ground foraging**	7.07E-07	5.12E-07	3.15E-07	4.59E-07	8.01E-07
**Foraging generalist**	1.97E-18	4.23E-17	1.39E-16	1.31E-16	4.66E-15
**Aquatic plunge**	5.09E-07	7.00E-07	2.32E-07	1.29E-07	3.27E-07
**Aquatic dive**	2.40E-07	2.29E-07	3.14E-07	5.22E-07	5.15E-07
**Arboreal gleaning**	6.78E-07	5.96E-07	4.08E-07	4.27E-07	7.64E-07
**Aerial screening**	2.05E-08	8.62E-09	2.02E-08	1.91E-08	4.01E-08
**Aerial sallying**	2.48E-07	2.10E-07	1.64E-07	1.84E-07	2.06E-07
**Bark gleaning**	4.81E-07	4.97E-07	1.29E-07	1.80E-07	3.30E-07
**Aquatic surface**	6.77E-14	2.64E-12	1.20E-13	1.41E-13	4.54E-13
**Non-flesh**	5.51E-07	4.83E-07	2.75E-07	4.29E-07	6.43E-07

**Flesh**	1.16E-07	1.14E-07	1.08E-07	8.32E-08	1.23E-07
**Non-aquatic**	3.91E-07	2.78E-07	1.95E-07	3.01E-07	3.60E-07
**Aquatic**	1.59E-07	3.17E-07	2.50E-07	1.19E-07	4.11E-07

Table S9. Evolutionary rates per vertebral region.

Groups	Observed Rate Ratio	Effect Size	C2 rate	C25% rate	C50% rate	C75% rate	Last rate	P
**Vertebra**	1.809	11.2123	3.15861E-07	2.82461E-07	2.02411E-07	2.42699E-07	**3.66155E-07**	0.000999001
