# Supplementary material for: The neck as a keystone structure in avian macroevolution and mosaicism
Source: BMC Biol. 2023 Oct 13;21:216. doi: 10.1186/s12915-023-01715-x (PMC10576348; doi:10.1186/s12915-023-01715-x)
Supplement: Supplementary file 2 — Additional file 2: Fig. S1. Landmarks scheme used for cervical vertebrae. Red landmarks are fixed (invariant and variant), gold landmarks are landmarks that form curves, blue landmarks are landmarks that form patches. [file 12915_2023_1715_MOESM2_ESM.pdf]

Additional File 2 for Marek & Felice 'The neck as a keystone structure in avian macroevolution and mosaicism'

Landmark scheme for cervical vertebrae

Invariant landmarks
1 - Maximum curvature of the dorsal and right part of the cranial articular surface
2 - Maximum curvature of the dorsal and left part of the cranial articular surface
3 - Maximum curvature of the dorsal and left part of the caudal articular surface
4 - Maximum curvature of the dorsal and right part of the caudal articular surface
5 - Junction of the vertebral arch and left prezygopophyseal facet
7 - Junction of the vertebral arch and right prezygopophyseal facet
9 - Anterior junction of the vertebral arch and right postzygopophyseal facet
10 - Posterior junction of the vertebral arch and right postygopophyseal facet
11 - Anterior junction of the vertebral arch and left postzygopophyseal facet
12 - Posterior junction of the vertebral arch and left postzygopophyseal facet
15 - Caudal maximum curvature of vertebral arch
16 - Cranial maximum curvature of vertebral arch
17 - Middle ventral curvature of the cranial articular surface
18 - Middle ventral curvature of the caudal articular surface
19 - Middle dorsal curvature of the cranial articular surface
20 - Middle dorsal curvature of the caudal articular surface

Variant landmarks
6 - Lateral most extent of the left transverse process
8 - Lateral most extent of the right transverse process
13 - Caudal most extent of the left costal process/rib
14 - Caudal most extent of the right costal process/rib

Landmarks forming curves
(1) 1-19 - Right dorsal outline of cranial articular surface
(2) 19-2 - Left dorsal outline of cranial articular surface
(3) 2-17 - Left lateral and ventral outline of cranial articular surface
(4) 17-1 - Right ventral and lateral outline of cranial articular surface
(5) 4-20 - Right dorsal outline of caudal articular surface
(6) 20-3 - Left dorsal outline of caudal articular surface
(7) 3-18 - Left lateral and ventral outline of caudal articular surface
(8) 18-4 - Right ventral and lateral outline of caudal articular surface
(9) 5-16-7 - Border of the dorsocranial face of the vertebral arch
(10) 11-15-9 - Border of the dorsocaudal face of the vertebral arch
(11) 10-4 - Border of the right ventrocaudal face of the vertebral arch
(12) 12-3 - Border of the left ventrocaudal face of the vertebral arch
(13) 5 - Outline of the left prezygopophyseal facet
(14) 7 - Outline of the right prezygopophyseal facet
(15) 11 - Outline of left prezygopophyseal facet
(16) 9 - Outline of right prezygopophyseal facet
(17) 15-16 - Central line along the dorsal face of the vertebral arch
(18) 17-18 - Central line along the ventral face of the vertebral body
(19) 19-17 - Central line along the cranial articular surface
(20) 18-20 - Central line along the caudal articular surface

Landmarks forming patches
1-19-2-17-1 - Cranial articular surface
4-20-3-18-4 - Caudal articular surface

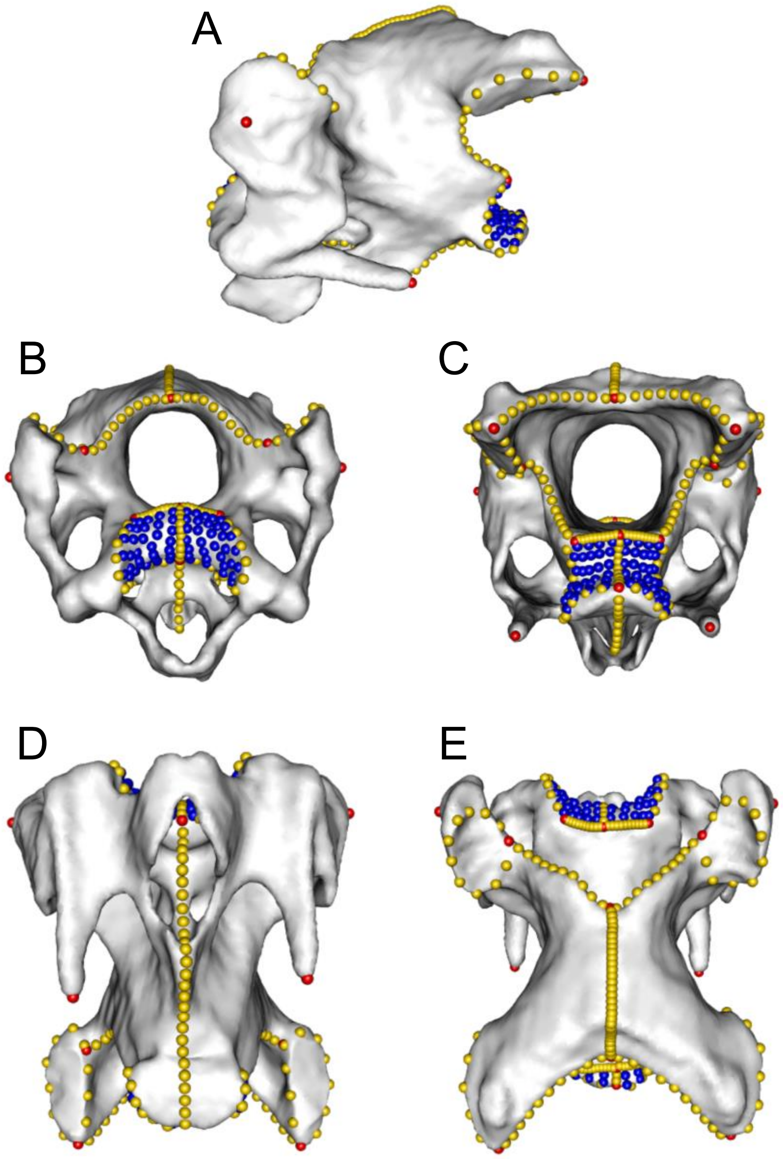

Figure S1. Landmarks scheme used for cervical vertebrae. Red landmarks are fixed (invariant and variant), gold landmarks are landmarks that form curves, blue landmarks are landmarks that form patches. Cervical vertebrae (C75%) of *Basilornis mirandus* in A) left lateral view, B) anterior view, C) posterior view, D) ventral view, E) dorsal view.
